# A systematic review of health state utility values for older people with acute myeloid leukaemia

**DOI:** 10.1007/s11136-024-03734-9

**Published:** 2024-08-22

**Authors:** Elise Button, Hannah Carter, Nicole C. Gavin, Thomas W. LeBlanc, Nikki McCaffrey

**Affiliations:** 1 Centre for Healthcare Transformation, Cancer and Palliative Care Outcomes, Queensland University of Technology, Level 7, Q Block, 66 Must Avenue, Kelvin Grove, Brisbane, QLD Australia; 2 Australian Centre for Health Services Innovation and Centre for Healthcare Transformation, School of Public Health and Social Work, Faculty of Health, Queensland University of Technology, Brisbane, QLD Australia; 3 Cancer Care Services, Metro North Hospital and Health Services, Queensland Health Brisbane, Brisbane, QLD Australia; 4 Duke University School of Medicine, Durham, NC USA; 5 Deakin Health Economics, School of Health and Social Development, Deakin University, Melbourne, VIC Australia

**Keywords:** Acute myeloid leukaemia, Older person, Health state utility value, Quality of life

## Abstract

**Purpose:**

Older people with acute myeloid leukaemia (AML) have a poor prognosis, reduced health-related quality of life (HRQoL) and require substantial healthcare resources. The objectives of this systematic review were to determine what health state utility values (HSUVs) are reported in the literature that can be used in economic evaluations of interventions for older people with AML, identify research gaps, and discuss directions for future research.

**Methods:**

The following databases were searched for studies published from inception until Feb 2023: PubMed, EMBASE, CINAHL, PsycINFO, Cochrane, and EconLit. Studies were included if they reported on HSUVs of people with AML >60 years, or HRQoL data that could be mapped to HSUVs using currently published algorithms.

**Results:**

Of 532 studies identified, 7 met inclusion (4 full studies and 3 conference abstracts). Twenty-eight potentially eligible studies were excluded as they did not report HRQoL measures in sufficient detail to be mapped to utility values. Included studies reported on health states of newly diagnosed disease (n=4 studies), intensive therapy (n=1 study), controlled remission (n=3 studies), and relapsed or refractory disease (n=2 studies). No studies reported on low intensity therapy or supportive care health states. Utility values were largely reported via the EuroQol and ranged from 0.535 (intensive therapy) to 0.834 (controlled remission).

**Conclusion:**

There are gaps in knowledge on HSUVs for older people with AML, particularly for certain treatment-related health states. Future articles should publish comprehensive HRQoL outcomes to enable use in economic evaluation.

**Supplementary Information:**

The online version contains supplementary material available at 10.1007/s11136-024-03734-9.

## Abstract (summary)

***Why is this study needed?***

Certain types of aggressive leukaemia more commonly occur in older people. The treatments for aggressive leukemia in older people are changing rapidly, are expensive, and can lead to reduced quality of life.

***What is the key problem/issue/question this manuscript addresses?***

It is important for researchers and clinicians to have data on the quality of life of older people with aggressive leukaemia so they can compare the different treatment options and understand which provide the best ‘value’ or cost-effectiveness in terms of increasing both duration and quality of life relative to the resources consumed. These studies are commonly referred to as ‘cost-utility analyses’.

***What is the main point of your study?***

This review aimed to gather and summarise the published data on quality of life of older people with aggressive leukaemia that could be used in cost-utility analyses of different treatments. We also wanted to identify where that was insufficient data and suggest where future work is needed.

**What are your main results and what do they mean?**

We found seven studies reporting quality of life data that could be used in evaluations exploring cost-effectiveness of different treatments for older people with aggressive leukaemia. We found that many studies collected quality of life data but didn’t report it in a way that could be applied within cost-utility analyses. This represents a missed opportunity for use of the data in future work or in healthcare decision making. Researchers should be encouraged and supported to publish HRQoL measures in sufficient detail to be applied in cost-utility analyses.

**Supplementary Information:**

The online version contains supplementary material available at 10.1007/s11136-024-03734-9.

## Introduction

Acute myeloid leukaemia (AML) is an aggressive haematological malignancy that affects the bone marrow and blood [1]. In the USA alone, over 21,000 are diagnosed with AML each year [2] with global incidence increasing [3]. Although AML can occur at any age, the disease most commonly develops in people over the age of 65 years [4] with a median age of 68 at diagnosis [5]. The term ‘older’ is used in the literature to describe people who are greater than 60–65 years of age [5, 6]. Older people with AML have a poorer prognosis compared to younger cohorts as they often have secondary disease arising from a prior blood disorder or cancer treatment, unfavourable cytogenetics, reduced physical performance status, and comorbidities [7, 8]. Treatment of older people with AML is particularly challenging as this cohort are highly susceptible to their underlying disease and treatment related toxicity, requiring a unique focus in research, guidelines, and clinical care [9].

Treatment pathways for AML are dependent on patient prognosis and ability to tolerate treatment [10]. These pathways include intensive therapy (i.e., aggressive chemotherapy +/- stem cell transplantation), low intensity therapy (i.e., low dose chemotherapy and immune therapies/targeted therapies), and supportive care (i.e., blood products and antimicrobials) [10]. All treatment pathways are associated with fatigue, poor performance, anxiety, depression, and reduced health related quality of life (HRQoL) [8, 11] Intensive therapy results in treatment-related symptoms and toxicities [6], while complications and symptoms associated with low intensity therapy and supportive care are often related to the underlying disease [8]. Treatments can extend over many months or even years, require frequent hospital visits, and be associated with significant toxicities and complications [8].

Although AML is most often diagnosed in older people, many older people are unable to receive the ‘standard’ treatment of intensive therapy [6]. Intensive therapy with curative intent is often recommended for younger people, and is associated with considerable mortality and morbidity in older people [6]. Many older people or those who are not candidates for intensive therapy are offered low intensity therapy, charactered by reduced toxicities but still with the intent of prolonging life, or supportive care aimed to control symptoms [6]. Intensive therapy, low intensity therapy, and supportive care for people with AML is associated with significant healthcare utilisation and is resource intensive [12, 13]. Major drivers of costs include hospitalisation and medication costs [14, 15]. Costs of treatment are expected to increase with the development of novel therapies [14]. The past few years have seen the treatment landscape of older people with AML change rapidly with many novel targeted therapies approved for use in the USA and internationally, all associated with improved efficacy but also considerable toxicities and costs [16]. Researchers are tasked with evaluating the most effective and cost-effective of many new treatment options for older people with AML.

Economic evaluation is increasingly relied upon to inform decision-making on how society should allocate scarce healthcare resources to best meet needs [17]. Cost-utility analyses are considered the gold standard form of economic evaluation [18]. These analyses adopt the quality adjusted life year (QALY) as the measure of effectiveness. Health state utility values (HSUVs) represent an individual’s preference for being in a particular health state and are used to calculate QALYs by weighting the time spent in a given health state by the HRQoL of that health state [19]. Health state utility values are usually obtained through asking the opinions of the general public to imagine what it would be like to live in different scenarios of health and are defined on a scale from 0.0 (death) to 1.0 (perfect health). Health economists and researchers rely on HSUVs (often derived from systematic reviews) to inform the parameters of model-based economic evaluations to evaluate healthcare interventions [20].

Limited previous work has focused on HSUVs of older people with AML to inform economic evaluation, despite their unique clinical trajectory, poor quality of life and prognosis, and resource intensive treatment. A 2018 systematic review by Forsythe and colleagues reported HSUVs for people with AML of any age, with a search strategy covering publications until 2016 [21]. Other reviews have focused more broadly on HRQoL of measures for people with AML who were ineligible for intensive therapy [8] (search conducted until 2017), or patient reported outcome measures for people with AML and myelodysplastic syndromes (search conducted up until 2019). These reviews were not focused on data that can be used in economic evaluation. A catalogue of HSUVs (derived from Euro-Qol data) of all haematological malignancies was published in 2020, with a search conducted until 2018) with one study focused on older people with AML [22]. These reviews are outdated in consideration of the rapid changes in treatments over the past several years for older people with AML. Generic catalogues of EuroQol-Five Dimension (EQ-5D) of people with chronic illness do not present data specifically on older people with AML in various relevant health states [23, 24]. An up-to-date systematic review is therefore needed, gathering HSUVs of older people with AML to understand data that is readily accessible, to inform cost-utility analyses comparing the many new treatments available for older people with AML.

Therefore, the aims of this review were to systematically review the literature on published HSUVs for older people with AML to determine data currently available to inform economic evaluations, identify research gaps, and discuss directions for future research.

## Methods

### Protocol and registration

A systematic review of the literature was conducted to identify articles reporting HSUVs of older people with AML in sufficient detail to be used in economic evaluations. The review was conducted and reported according to the PRISMA (Preferred Reporting Items for Systematic Reviews and Meta-Analyses) checklist and flow diagram [25]. Published guidelines on conducting systematic reviews of HSUVs informed the review to enhance quality, rigor, and reproducibility [20, 26, 27]. The protocol was registered prospectively with PROSPERO (CRD42022301344).

### Search strategy

A broad and extensive search was conducted of electronic databases covering medical, nursing, and economic literature, including PubMed, EMBASE, CINAHL, PsycINFO, Cochrane (Database of Systematic Reviews and Registry of Controlled Trials databases), and EconLit for studies published from inception until February 2023. This broad time period was used to ensure no potentially relevant publications were missed. Forward searching of citations was conducted in Google Scholar as well as hand searching of reference lists of included studies. ProQuest Dissertation and Theses Database was searched for grey literature.

### Eligibility criteria

Eligibility criteria for the systematic review are presented in Table 1. Studies were included if they reported on HSUVs of people with AML aged 60 years or older. This age was chosen so no potentially relevant studies were missed, as the literature defines ‘older’ as 60–65 years and over. Studies that reported data all age groups of people with AML, or multiple cancers were included if HSUVs for older people with AML were reported separately. If data were not reported separately, at least 80% of the sample had to be older people with AML to be included. This age restriction was applied to capture HSUVs that accurately reflected the experience of older people with AML, who have unique issues compared to younger people.

**Table 1:** Selection criteria

Element	Inclusion	Exclusion
Patient population	*≥* 60 years People with AML (de-novo or secondary)	People with other types of haematological malignancies
Intervention	All, including no intervention	Any not listed in inclusion criteria
Comparator	All, including no comparator	Any not listed in inclusion criteria
Outcome measure	Utilities, disutilities, or QALYs derived from direct or indirect methods Only multi-attribute utility instruments HRQoL reported in validated measures that could be converted to utility	Utilities reported from a secondary reference source Single-attribute utility instruments (i.e., visual analogue scale)
Study design	Primary research in English language, any date that reported HSUVs Report the method and source of obtained utility values	Reviews Editorials Notes/Comments/Letters

HRQoL = health-related quality of life; HSUVs = health state utility values; QALYs = Quality adjusted life years

Studies reporting HRQoL in sufficient detail to be converted to utility values with published algorithms were also eligible (HRQoL terms displayed in Supplementary file 1 – indirect valuation methods). Currently available published algorithms for HRQoL measures were sourced from a recent systematic review focused on HSUVs of people with a cancer diagnosis [28]. Studies that reported the EQ-5D Visual Analogue Scale alone (and not the individual domain responses or overall utility scores) were excluded. Published conference abstracts were included if they reported sufficient information on HSUVs and other eligibility criteria could be sourced from original publications (i.e., age of sample). Three relevant systematic reviews were used for reference cross-checking but not included [8, 21, 29].

### Search terms

A combination of key terms and synonyms, and Medical Subject Headings (MeSH) were developed, guided by the current related literature [8, 20, 21], and tailored to each electronic database (see Supplementary file 1 for search terms). Database search terms and results are displayed in Supplementary files 1 and 2.

### Study selection

Title, abstract and full-text screening of studies was conducted by two reviewers independently (EB, NG) against selection criteria in Covidence online systematic review software [30]. Ambiguities were discussed with other members of the research team (NM, HC).

### Data extraction

Data were extracted independently by two reviewers (EB, NG) using a data extraction form based on the relevant literature and guidelines [20, 21, 27] and included publication details (year published, country), study aim, design, years of data collection, intervention and control (if applicable), sample (number characteristics of participants), age, description of HSUVs (measure of valuation and tariff), and the health state and utility values reported (via mean or median, and variance if reported). If feasible, HRQoL measures were extracted and converted to HSUVs using published algorithms. A range of non-preference based HRQoL scores can be converted to utility values including the Quality of Wellbeing Scale, Short Form Health Survey, Assessment of Quality of Life, and the Health Utilities Index [31]. The EORTC-QLQ-C30 is the most widespread HRQoL tool used in the oncology and AML literature that can be mapped to utility values [21, 29].

### Quality assessment

Quality assessment of included studies was assessed by two independent reviewers (EB, NG) using guidelines on searching and selection of HSUVs from the literature (developed from the NICE [National Institute of Clinical Excellence] Decision Support Unit Technical Support Document 9) [32]. These guidelines provide an overview of the key criteria to assess quality of data including the following: (1) sample size; (2) respondent selection and recruitment; (3) inclusion/exclusion criteria; (4) response rates to instrument used; (5) loss to follow-up; (6) missing data; (7) any other problems with the study; and 9) appropriateness of measure [32]. This method was chosen as it has been commonly used in other systematic reviews of HSUVs [33, 34], is easy and practical to use, and covers the key quality domains in the International Society Pharmaceutical Outcomes Research guidance on reviewing the literature on HSUVs [35]. Further quality assessment was performed for completeness, using the relevant CASP (Critical Appraisal Skills Programme) checklist [36]. These checklists were selected as they provide a clear, systematic, consistent approach to assess quality of various study designs [36].

### Data synthesis

Utility values were mapped against health states (see Table 2) according to the clinical characteristics of the study sample. These health states were developed based on clinical knowledge and published literature, and their validity was confirmed via expert review (two experienced haematologists [JB, TL], and two senior haematology nurses [AT, NG]. Due to the limited number of studies included in the review and differences between the studies in population and interventions evaluated, pooling data was considered inappropriate [20]. Additional data were extracted from studies with HRQoL measures reported in sufficient detail to be mapped to a relevant HSUV. Mapping algorithms were selected for the relevant HRQoL instruments once selection of studies was complete [37].

**Table 2:** Health state utility values of older people with acute myeloid leukaemia

Health state 1	Newly diagnosed acute myeloid leukaemia
Health state 2	Intensive therapy
Health state 3	Low intensity therapy
Health state 4	Supportive care
Health state 5	Controlled remission
Health state 6	Relapsed or refractory acute myeloid leukaemia
Health state 7	Death

## Results

### Search results

**Fig. 1:**
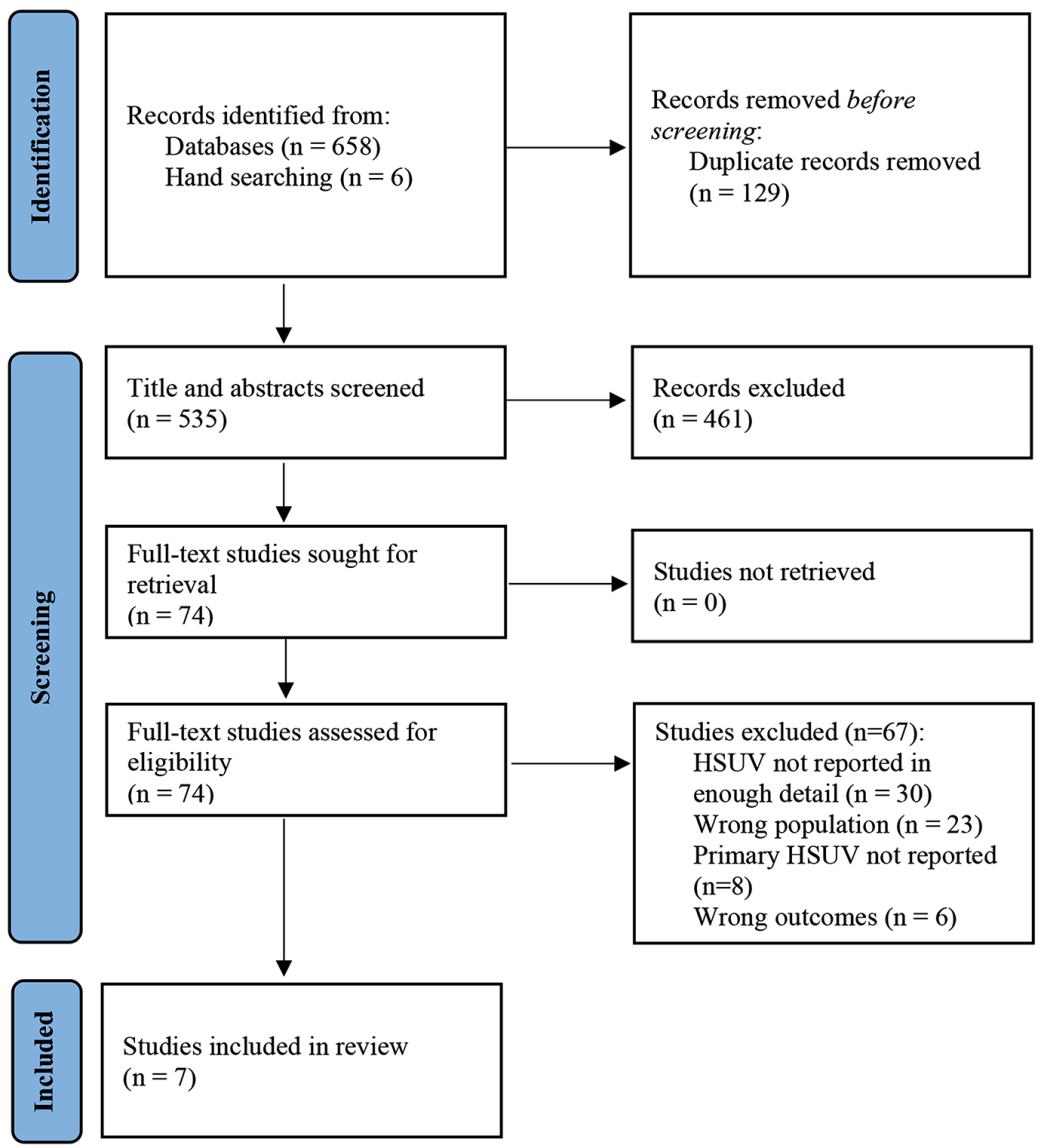
PRISMA flow diagram

Database (*n* = 658) and hand searching (*n* = 3) identified 532 studies, following removal of duplicates. After screening, seven studies were included in the review (see Fig. 1. PRISMA flow diagram, and Supplementary file 4. Studies excluded after full-text review). Of note, 28 potentially eligible studies were excluded as they did not report HRQoL measures in sufficient detail to be mapped to utility values.

### Characteristics of studies

The seven studies included: one economic evaluation using data from a randomised clinical trial [38], two observational studies gathering HRQoL measures [39, 40], and four studies that used data derived from randomised clinical trials (reported data of entire sample) to validate a HRQoL tool [41] or describe HSUVs of patient cohorts [42–44]. Characteristics of the included studies are presented in Table 3. Six studies included newly diagnosed patients [38, 40–44], and one included people at any stage of the illness [39]. The combined total sample was 2,606 people, with ages ranging from median 61 (IQR 49–69) [40] to mean 76 years (range 41–91) [43]. One study included a mixed sample of people with acute leukaemias of various types, and reported data on people with AML separately [39].

**Table 3:** Characteristics of, and health state utility values reported, in included studies

Author, year, country	Study aim	Design, years of data collection	Intervention, control	Sample	Age	Valuation method (tool and tariff)	Health states and utility values
*Full studies*							
Groot 1998 [1] Netherlands	To compare effects and costs of GM-CSF as an adjunct to intensive chemotherapy in elderly patients with AML	Cost-effectiveness study based on prospective, randomised, multi-centre clinical trial (societal perspective) – survival, remission and HRQoL measured (Nov 1990 – Oct 1994)	Intervention: GM-CSF + daunomycin cytosine arabinoside (*n* = 157) Control: Daunomycin cytosine arabinoside (*n* = 161)	> 60 with untreated newly diagnosed *de novo* or secondary AML (*N* = 318) part of Phase III study	Intervention 60–70 58% 70–80 39% > 80 3% Control 60–70 64% 70–80 33% > 80 3%	EuroQol (tariff not reported)	*Control v intervention* Start of induction: 0.706/0.648 (mean 0.677 of control and intervention) During hospitalisation (day + 2 after treatment): 0.671/0.535 After hospitalisation (at home): 0.727/0.680 6 months post-treatment: 0.844/0.806 12 months post-treatment: 0.750/0.744 24 months after treatment: 0.813/0.595 *Remission v no controlled remission* 6 months post-treatment: 0.872/0.689 12 months post-treatment: 0.804/0.563 24 months post-treatment: 0.813/0.595
Mamolo 2019 [2] USA	To characterise demographic, clinical, symptoms and HRQoL in adults with newly diagnosed or relapsed or refractory *de novo* AML	Multi-site cross-sectional survey – 2 HRQoL tools delivered to consecutive patients accessing care (Feb – May 2015)	Nil	People with newly diagnosed *n* = 339, 87% or relapsed or refractory *n* = 50, 13% *de novo* AML in community receiving ‘routine’ care (*N* = 389) Intensity of treatment: high *n* = 181, 53% and low *n* = 158, 47%	Median age: entire sample 61 (IQR 49–69), newly diagnosed 61 (IQR 49–69), relapsed or refractory 56 (IQR 48–66) – data not reported on relapsed or refractory group	EQ-5D-3 L USA values	Newly diagnosed: mean 0.74 (SD 0.21), median 0.81 (IQR 0.59–0.84)
Lennmyr 2020 [3] Sweden	To report on the feasibility and results of collection of patient reported outcome registry data	Prospective pilot of cross-sectional survey of people with acute leukaemia (April 2014 – Dec 2016) – 1 HRQoL tool and 2 patient health questionnaires sent out 6 months post diagnosis	Nil	People with acute leukaemia: AML *n* = 212, 83% Complete remission *n* = 149, 93%	Median age: entire sample 64 (range 18–90), AML 65 (range 21–90)	EORTC-QLQ-C30 (data converted to EQ-5D UK values)	6 months post diagnosis (+ median 58 days, range 10–316) AML group: 0.834 (Estimated using algorithm reported by Kim et al. 2012 [4])
Piepert 2020 [5] USA	To validate the FACT-Leu in patients with AML who are not candidates for intensive therapy	Prospective observational study – completion of 3 HRQoL tools every 28 days until completion of treatment (Aug 2015 – Feb 2018) Patients enrolled in Phase 2/3 study	Intervention: Decitabine + talocotuzuma Control: talocotuzuma only (EQ-5D data provided for entire cohort)	Patients with AML not eligible for intensive chemotherapy (*N* = 317)	Mean age 75 (range 51–92)	EQ-5D-5 L UK values	Day 1 of non-intensive chemotherapy cycle: 0.67 (SD 0.26, min − 0.36, max 1.00, median 0.71), 25th percentile 0.57, 75th percentile 0.85 Utility values for other time points not reported.
*Abstracts*							
**Author, year, country**	**Study aim**	**Design, years of data collection**	**Intervention, control**	**Sample**	**Age**	**Valuation method (tool and tariff)**	**Health states and utility values**
Pierson 2017 [6] International	To evaluate physical, psychological and social functions in newly diagnosed AML ineligible for aggressive therapy	Data drawn from Phase 3 RCT (*DACO*-016) at baseline (data presented for entire cohort). Jan 2006 – April 2009	Intervention: decitabine Control: physician’s choice of either supportive care or low-dose cytarabine.	Newly diagnosed people with AML ineligible for aggressive therapy (*N* = 485)	Mean age of 73.2 years; a median of 73 years and interquartile range (69–77)	EORTC-QLQ-C30 (data converted to EQ-5D UK values)	Baseline All patients: 0.81 ECOG PS 0: 0.88 ECOG PS 1: 0.81 ECOG PS 2: 0.75 (Estimated using algorithm reported by Kim et al. 2012 [4])
He 2018 [7] International	To evaluate HRQoL in a cohort of patients with AML who were ineligible for aggressive therapy	Data drawn from phase 2/3, parallel design RCT (AML 2002) at baseline 2015–2018	Intervention: decitabine and talacotuzumab Control: decitabine and placebo (data presented for entire cohort)	Patients with AML not eligible for aggressive therapy (*N* = 309)	Mean age 74.9 years, > 69 81%	EQ-5D-5 L	Mean baseline: 0.71 (cycle 1, day 1)
Pratz 2022 [8] International	To estimate the health state utility values among newly diagnosed people with AML ineligible for aggressive therapy	Data from clinical trials: Phase 3, multicentre, double-blind, RCTs (Viale-A and Viale-C) that collected EQ-5D data for patients in different health states Feb 2017 – May 2019 (Viale A) [9] Oct 2014 - data cut off July 2017 (Viale C) [10]	*Viale A* Intervention: azacitidine and venetoclax Control: azacitadine and placebo. *Viale C* Intervention: venetoclax and decitabine. Control: azacytidine and placebo. (Pooled data presented for both studies)	Total of 576 patients (390 + 186) ineligible for aggressive therapy	Median 76 (range 41–91), *≥* 75 61% - (Viale A trial data) [9] Median 74 (range 68–85), > 75 44% (Viale C trial data) [10]	EQ-5D utility scores were estimated based on the pooled data from all patients and calculated using UK value sets (other countries not reported separated so not listed here)	*UK values* Event free survival with controlled remission: 0.747 (SE = 0.013). Event free survival without controlled remission: 0.725 (SE = 0.014). Progressive disease or relapsed disease: 0.628 (SE = 0.017).

AML – acute myeloid leukaemia, QoL – quality of life, GM-CSF – granulocyte-macrophage colony-stimulating factor; EORTC-QLQ-C30 – European Organisation for Research and Treatment of Cancer Quality of Life Questionnaire-C30, HRQoL – health-related quality of life, EQ-5D – Euro-Qol-5 Dimensional 3 or 5 Level, SD – standard deviation, IQR – inter quartile range; RCT – randomised controlled trial; SE – standard error; ECOG PS – Eastern Oncology Outcomes Group Performance Status

### Health state utility values

The HSUVs reported in the included studies are displayed in Table 3 and mapped to health states in Table 4. Four studies measured HSUVs via the EQ-5D [40, 41, 43–45] and one via the earlier EuroQol (no further description provided) [38]. Two studies reported European Organization for Research and Treatment of Cancer Quality-of-Life Questionnaire Core 30 (EORTC-QLQ-C30) data [39, 42]. The lowest HSUV (0.535) was reported for the health state of intensive therapy (day 2 after chemotherapy) and the highest HSUV (0.834) for people in controlled remission (6 months post-treatment) [39]. A ‘General AML state’ was used to categorise HSUVs where the health state of the sample was unclear. Data from two studies were mapped to in the ‘general AML state’: (1) Mamalo and colleagues reported data from people who were newly diagnosed or relapsed (with no description of treatment) ; and (2) Groot and colleagues reported data from people at home after hospitalisation (after daunomycin cytosine arabinoside) with the time frame not described [38]. Values ranged from 0.68 to 0.81 in this state (Table 3). No studies were found reporting HSUVs for low intensity therapy or supportive care, four reported HSUVs for newly diagnosed disease, five studies reported on the health states of controlled remission, and relapsed or refractory disease (see Table 4). Of note, utility values reported at baseline (day 1 or prior to chemotherapy) were categorised as newly diagnosed disease as they did not reflect treatment health states.

**Table 4:** Health states of older people with acute myeloid leukaemia

Health state	Health state described in study	Utility value point estimate	Reference	Quality score +	Data source
Newly diagnosed AML	Prior to starting chemotherapy	Mean 0.677*	Groot 1998 [1]	Low	Euro-Qol
	Prior to starting chemotherapy	Mean 0.67 Median 0.71	Piepert 2020 [5]	High	EQ-5D-5 L
	Baseline – day 1 of chemotherapy	Mean 0.71	He 2018 [7]	Mod	EQ-5D-5 L
	Prior to starting chemotherapy	All patients: 0.81 ECOG PS 0: 0.88 ECOG PS 1: 0.81 ECOG PS 2: 0.75	Piereson 2017 [6]	Mod	EQ-5D
Intensive therapy	Day + 2 after daunomycin cytosine arabinoside +/- GM-CSF (control/intervention)	Mean 0.671/0.535	Groot 1998 [1]	Low	EuroQol
Low intensity therapy	N/A	-	-		-
Supportive care	N/A	-	-		-
Controlled remission	6 months post chemotherapy	Mean 0.872	Groot 1998 [1]	Low	Euro-Qol
	6 months + median 58 days (range 10–316) from timeframe (93% complete remission)	**0.834	Lennmyr 2020 [3]	Low	EQ-5D
	12 months post chemotherapy	Mean 0.804	Groot 1998 [1]	Low	Euro-Qol
	24 months post chemotherapy	Mean 0.813	Groot 1998 [1]	Low	Euro-Qol
	Event free survival with controlled remission	0.747 (SE 0.013)	Pratz 2022 [8]	Mod	EQ-5D
Relapsed or refractory AML	6 months post chemotherapy	Mean 0.689	Groot 1998 [1]	Low	Euro-Qol
	12 months post chemotherapy	Mean 0.563	Groot 1998 [1]	Low	Euro-Qol
	24 months post chemotherapy	Mean 0.595	Groot 1998 [1]	Low	Euro-Qol
	Event free survival without controlled remission	0.725 (SE 0.014)	Pratz 2022 [8]	Mod	EQ-5D
	Progressive or relapsed disease	0.628 (SE 0.017)	Pratz 2022 [8]	Mod	EQ-5D
General AML state	Newly diagnosed or relapsed - median 4 (IQR 2–9) and mean 7 (SD 8.2) months from ‘diagnosis’, treatment not described	Mean 0.74, median 0.81	Mamolo 2019 [2]	Low	EQ-5D-3 L
	After hospitalisation (at home) after daunomycin cytosine arabinoside – time frame not described (control/intervention)	Mean 0.727/0.680	Groot 1998 [1]	Low	EuroQol

*Mean utility of control and intervention group

**Estimated using algorithm by Kim et al., 2012 [4]

+Quality score based on authors’ assessment overall quality of study (see Table 5)

GM: CSF – granulocyte-macrophage colony-stimulating factor, AML – acute myeloid leukaemia, EQ-5D – Euro-Qol-5 Dimensional 3 or 5 Level, ECOG PS – Eastern Cooperative Oncology Group Performance Score, SE - standard error

The only studies in the review which included convertible HRQoL measures administered the EORTC-QLQ-C30 [39, 42]. To allow for conversion, data on the individual domains (i.e., physical functioning, pain, global health status) within the EORTC-QLQ-C30 had to be reported, as opposed to an overall summary score. A published algorithm by Kim et al. (2012) in a mixed cancer population receiving chemotherapy was used to map EORTC-QLQ-C30 scores to EQ-5D utility values [46]. This algorithm was selected as it was developed in a population with similarities to the review population [37] and led to clinically plausible utility values (comparable to other utility values for the same health state). Calculations are shown in Supplementary file 3. Of note, no variance was available when HSUVs were converted via algorithms.

### Quality assessment

Quality assessment of included full studies and abstracts using updated guidance from the NICE Decision Support Unit Technical Support Document 9 [32] is displayed in Table 5. The authors’ overall assessment of quality (as determined in Table 5) is included in Table 4, to provide guidance on the level of confidence in each of the HSUVs. All studies had relatively large sample sizes (*n* = 212–576). Three studies were subjectively deemed by the authorship team to have low quality, relating to low response rates (6–52%, 15% [40]) and poorly described health states [38–40]. One study was considered high quality due to the high response rate and broad selection criteria, and HSUVs were reported on a clearly defined health state. Of note, only two studies reported data from the broader population [39, 40], rather than drawing from clinical trials with stringent eligibility criteria. Where there were insufficient data to determine methodological quality of abstracts, related publications were sourced that reported relevant information in full (noted with reference in Table 5). The three abstracts that were included in the review [42–44] were of moderate quality with clearly defined health states and good response rates, but had stringent selection criteria with data were drawn from RCTs.

**Table 5:** Quality assessment of included studies based on updated guidance from NICE TDS

Study	Sample size	Respondent selection and recruitment	Inclusion/exclusion criteria	Response rates to instrument used	Loss to follow-up	Missing data	Any other problems with the study	Appropriate-ness of measure	Authors’ assessment overall quality
*Full studies*									
Groot 1998 [1]	*N* = 318	Data drawn from prospective, randomised, multi-centre clinical trial. 326 people registered, 8 ineligible (reasons provided).	Inclusion: >60 with untreated newly diagnosed *de novo* or secondary AML who enrolled in clinical trial	EuroQol response rate: *Control v intervention* Start of intervention: 6%/7% During hospitalisation: 12%/6% After hospitalisation: 9%/5% 6 months post-treatment: 37%/35% 12 months post-treatment: 52%/17% 24 months post-treatment: not reported	Not clearly described except if death was cause	Approach to missing data not described.	*Control v intervention groups* Performance status: 0 20%/20%, 1 30%/27%, 2 10%/14%, 3 1%/3%. Measure of variability: not reported.	EuroQol appropriate (at time of publication)	Low – low response rate, variation in response rates between control and intervention and at different time points, poorly described health states.
Mamolo 2019 [2]	*N* = 389 (*n* = 339, 87% newly diagnosed, 13% relapsed or refractory)	Data drawn from Adelphi Real World AML Disease Specific Programme. Survey invitations sent via physicians caring for people with AML in community setting. Consecutive 6–8 patients invited to participate	Inclusion: diagnosis of *de novo* AML, were currently receiving or had previously received active treatment for AML, aged ≥ 18 years old Exclusion: participating in a clinical trial	EQ-5D-3 L response rate 17% (entire sample)	N/A – one time point only	Missing EQ-5D data not discussed – low response rate of 17% to HRQoL questionnaire. Minimal missing patient characteristics.	Data collected newly diagnosed or relapsed people median 4 (IQR 2–9) and mean 7 (SD 8.2) months from diagnosis (unclear what health state in). ECOG (newly diagnosed − 0 36%, 1 35%, 2 23%.	EQ-5D-3 L appropriate	Low – low response rate, poorly defined health state, broad selection criteria.
Lennmyr 2020 [3]	*N* = 255 (AML *n* = 212, 83%, ALL *n* = 43, 17%)	All people in acute leukaemia registry (Swedish Cancer Registry) sent survey invitation	Nil specific – invitation sent out 6 months post diagnosis so people beyond initial ‘crisis’ of disease	EORTC-QLQ C30 response rate 64%. Responders were older than non-responders: median age 64 v 54 (range 18–90 v 18–89) respectively.	N/A – one time point only	Missing data for EORTC-QLQ-C30 1% (entire sample). Minimal missing data on characteristics of patients. Missing data for remission status 12% for people with AML.	Time from diagnosis: 6 months + median 58 days (range 10–316). Relatively good World Health Organisation Performance Status: AML 0–1 85%, 2–3 15%.	EORTC-QLQ C30 appropriate	Low – moderate response rate, poorly described health state
Piepert, 2020 [5]	*N* = 317	Data drawn from a 2-arm RCT of *N* = 326 people with AML who were unsuitable for intensive chemotherapy	Inclusion: ≥75 years or ≥ 65 years + several medical comorbidities or evidence of frailty	307 of 317 completed EQ-5D, 97% response rate	N/A – one time point only	Missing EQ-5D data not discussed – not likely to be an issue with EQ-5D reported for one time point with response rate of 97%.	HSUV data reported at one time point although collected at 28-day cycles. Mostly non-Hispanic white (85%), ECOG (19% 0, 43% 1, 38% 2)	EQ-5D-5 L appropriate	High – good response rate, broad selection criteria, clearly defined health states
*Abstracts*									
**Study**	**Sample size**	**Respondent selection and recruitment**	**Inclusion/exclusion criteria**	**Response rates to instrument used**	**Loss to follow-up**	**Missing data**	**Any other problems with the study**	**Appropriateness of measure**	**Authors’ assessment overall quality**
Pierson 2017 [6]	*N* = 485	Data drawn from a Phase 3 RCT of people ineligible for aggressive therapy (DACO-016)	Inclusion: >65 years, newly diagnosed AML, poor and intermediate risk cytogenetics. Exclusions: other cancers, previous treatments, candidate for transplant [11]	485 recruited, 454 completed, completed HRQoL questionnaire, 93.6% response rate	N/A as baseline data only	No missing data on characteristics of sample. 93.6% response rate at baseline	Abstract, reports minimal data (some data sourced from published study)	EQ-5D appropriate	Moderate - clearly defined health state, good response rate, stringent selection criteria
He 2018 [7]	*N* = 309	Data drawn from a Phase 2/3 parallel design RCT in people who were not eligible for aggressive therapy (AML 2002)	Inclusion: >75 or > 65 with comorbidities, previously untreated AML, ineligible for induction [12]	326 people recruited and 309 completed (94.8% response rate) [12]	N/A as baseline data only	No missing data on characteristics of sample. 94.8% response rate at baseline	Abstract, reports minimal data (some data sourced from published study)	EQ-5D-5 L appropriate	Moderate - clearly defined health state, good response rate, stringent selection criteria
Pratz 2022 [8]	*N* = 576 (*n* = 390 + *n* = 186)	Data drawn from 2 international clinical trials of newly diagnosed people with AML ineligible for intensive therapy (Viale A and Viale C)	Inclusion Viale A: >18, untreated AML, ineligible standard induction (older age or comorbidities). Exclusions Viale A: previously treated AML, favourable cytogenetic risk [9] Inclusion Viale C: *≥* 65 years at diagnosis and same as above for other criteria [10]	Response rate not reported in abstract – sample size in abstract does not match sample size in published paper due to publication at different cut-off date	Not reported in abstract	No missing data on characteristics of sample. Unclear if missing EQ-5D data.	Abstract, reports minimal data, discrepancies between abstract and published study data limits quality assessment	EQ-5D appropriate	Moderate - moderately defined health states, stringent selection criteria, response rates and missing data unclear

Key criteria to consider in quality assessment of HSUV studies [13]

*Sample size*: Precision of the estimate should be reflected in the variance around any estimate used in a model.

*Respondent selection and recruitment*: Does this results in a population comparable to that being modelled?

*Inclusion/exclusion criteria*: Does this result in a population comparable to that being modelled?

*Response rates to instrument used*: Do these exclude any individuals that might (e.g. the very elderly > 80 years old are often not included in studies)?

*Loss to follow-up*: Are response rates reported and if so, are the rates likely to be a threat to validity?

*Missing data*: How large is the loss to follow-up and are these reasons given? What are these likely to threaten the validity of estimates?

*Any other problems with the study*: Relevance of location (e.g., if patient recruited in different country to proposed model).

*Appropriateness of measure*: Is the measure used valid in the group of patients?

AML – acute myeloid leukaemia, ALL – acute lymphoblastic leukaemia, EORTC-QLQ-C30 – European Organisation for Research and Treatment of Cancer Quality of Life Questionnaire-C30, HRQoL – health-related quality of life, EQ-5D – Euro-Qol-5 Dimensional 3 or 5 Level, RCT – randomised controlled trial; ECOG PS – Eastern Oncology Outcomes Group Performance Status

Quality assessment of included full studies using the relevant CASP checklist is presented in Supplementary 2. Assessment of abstracts was not performed due to inadequate information reported in abstract format. Of the full text studies, Groot and colleagues’ [38] cost-effectiveness study had significant limitations due to unclear research question, lack of sensitivity analysis, and limitations in generalizability of findings to other settings. Mamolo et al. [40] and Lennmyr et al. [39] were limited by lack of information on the specific characteristics of participants, and consideration of how such confounding could bias study results. Piepert and colleagues [41] study scored consistently well in quality assessment, with only limitation relating to unclear application of results to a broader population (outside of a RCT).

## Discussion

### Health state utility values reported

This review highlights a substantial lack of published studies with easily accessible and comprehensively reported data on HSUVs for older people with AML that can be used in cost-utility analyses, particularly for health states related to treatment. The only study that reported on treatment-related health states (intensive therapy) reported utilities for an outdated chemotherapy regimen that no longer reflects current practice for frontline induction therapy of people with AML [47]. Further, two studies reporting HSUVs had insufficient data to determine the associated health state, limiting the usefulness for economic evaluation [38, 40]. A key finding is that no HSUVs were found for health states of low intensity therapy or supportive care. Rather most studies collected data within clinical trials of new drug treatments. While HRQoL has been reported for patients for low intensity therapy or supportive care health states, this gap in the HSUV literature limits cost effectiveness evaluations which include less aggressive therapies or usual care as a comparator, despite this potentially valid ‘next best’ treatment option. Quality assessment scores can be used to guide selection of the most reliable and appropriate HSUVs where multiple exist in a single health state. Other aspects to consider when choosing HSUVs are focused on relevance assessment criteria including: comparability between study and model, appropriate instrument used to describe health states, HSUV taken elicited directly from patient, and appropriate valuation of changes in HRQoL (i.e., general population using choice-based methods) [32].

The HSUVs reported in the included studies appear to be clinically relevant with values reflecting the illness trajectory of people with AML. The HSUVs for newly diagnosed and intensive therapy were lower, in line with the increased symptom burden and complications related to the underlying disease and chemotherapy experienced in these health states (i.e., fatigue, shortness of breath, bleeding, infections, nausea) [48]. Utility values during controlled remission were highest, reflecting a time of clinical stability with fewer symptoms and complications, and improved HRQoL [48]. Relapsed or refractory disease HSUVs declined over time as people drew closer to the end of life and experienced increasing symptoms and complications related to advancing disease (i.e., bone marrow suppression, pain, fatigue) [49].

The HSUVs included in this review were predominantly from EuroQol-generated instruments. There have been concerns expressed that these instruments may not be sensitive enough to capture all relevant domains of health for older people with AML [50, 51]. While EQ-5D is the most frequently used preference-based, generic method of obtaining utility values, there is mixed evidence regarding the responsiveness of the tool to detect meaningful change in all conditions [52]. Consequently, condition-specific measures that cover more cancer-relevant domains such as cognitive function [41] and fatigue have been used alongside the EuroQol tools [50]. Although the EQ-5D has been demonstrated to be appropriate for measuring change in HRQoL in patients treated with a palliative approach, the tool may not capture changes in symptom burden and holistic impacts of disease, which are important to people near the end of life [51, 53, 54]. The EORTC-QLQ-C30 captures more cancer-relevant domains of health and has been shown to be valid and reliable in people with advanced cancer [55] and is commonly used for older people with AML [29].

The EORTC-QLQ-C30 has traditionally been the most common HRQoL measure used for people with AML in observational and intervention based research [29]. During screening, many potentially eligible studies were excluded as their reporting of EORTC-QLQ-C30 data did not allow for conversion to HSUVs (where data on the individual domains were required rather than an overall summary score). Additionally, reporting of EQ-5D Visual Analogue Scale data alone were not able to be used as a utility value. Two high quality international clinical trials that collected HRQoL measures as a secondary endpoint were excluded from the review for this reason [56, 57]. Although HRQoL data were reported to address their study research questions, it was not able to be extracted as a HSUV or converted to a HSUV if it was reported as a mean difference, hazard ratio, for a single domain (i.e., fatigue, as a global health score, or as a summary score. Furthermore, a recent cost-effectiveness analysis of older people with AML included unpublished utility values sourced from a pharmaceutical company, which were not publicly available and had not undergone peer review. This highlights a potential gap for researchers when seeking accessible HSUVs to be used in economic evaluation. This could be due to a possible lack of awareness in the research community around the value of, and how to report HRQoL data that can be used in economic evaluation. Additionally, challenges may exist getting HSUV data published in appropriate journals.

Inadequate and mixed reporting of HRQoL data is a widespread issue associated with considerable research waste [58, 59]. The 30 excluded studies due to insufficiently reported HRQoL data could have filled important gaps in knowledge for treatment-related health states in this review (these studies have been noted in Supplementary file 3). Contacting authors from individual studies and seeking access to data is time consuming, often results in no reply (or bounced back emails), and can lead to delays and challenges regarding data sharing [60–62]. Ideally, HSUV data should be easily accessible in publications or open access data repositories. Furthermore, it is argued that appropriate and meaningful HRQoL measures are often not adequately measured in haematology clinical trials, leading to missed opportunity to collect and report valuable data. Greater awareness is needed so that variable reporting of data does not limit the full impact of research work, and lead to redundant, biased, underpowered and repetitive studies [58, 59]. Clinical trials in haematology should prioritise HRQoL as an important outcome that reflects the patient’s experience, and can be used to conduct economic analysis.

Researchers need appropriate and validated published algorithms to enable mapping of HRQoL measures to HSUVs. In this review, only studies with currently available algorithms were included, limiting studies reporting HRQoL data in potentially relevant populations. For the included studies, careful selection of a published algorithm was required to ensure clinically plausible HSUVs were calculated. Initially a published algorithm developed on a sample of women with locally advanced breast cancer with relatively good health was proposed for conversion of the scores to HSUVs [63, 64]. The algorithm developed in this population had previously been used to map HRQoL to utility values in people with AML and the population in the study of older AML patients [39] had similar clinical characteristics to the population used to generate the algorithm [63, 64]. This is recommended as a guiding factor when selecting an algorithm for mapping [37]. However, this initial algorithm returned utility values that were lower than clinically expected or plausible. Therefore, an alternative published algorithm by Kim and colleagues in a mixed cancer population receiving chemotherapy was used to map utility values which produced clinically plausible results [46]. Ideally, primary HSUV data should be collected and reported to avoid such issues and potential inaccuracies in data. Where this is not feasible, appropriate algorithms must be available to convert HRQoL data.

This review expands on similar previous systematic reviews of people with AML [8, 21, 29] by including six subsequently published studies and abstracts, enabling researchers to efficiently access up-to-date HSUVs to be used in cost-utility analyses. The main focus of the review by Stauder and colleagues was to understand which patient reported outcomes are used to measure HRQoL, therefore individual studies were not discussed or referenced (only meta-data presented), limiting usefulness for economic evaluation [29]. The 2018 review by Forsythe and colleagues focused on HSUVs derived from primary and secondary data of people with AML of any age [21].

### Implications and recommendations for future studies

The few studies included in this review provide valuable HSUVs that can be used in economic evaluations of interventions for older people with AML. However, the quality of the included studies varied, potentially limiting the usefulness of certain utilities; one study was outdated [38], another had a large amount of missing data [39], and insufficient details were provided to allow mapping of a HSUV to a health state [40]. The low number of included studies should be a driver for future research of older people with AML. The results demonstrate that more research is needed reporting HSUVs of older people with AML, particularly in treatment-related health states. Due to paucity of data, and low quality research, high quality studies reporting HSUVs for the following health states are desperately needed: (1) intensive therapy; (2) low intensive therapy; (3) and supportive care. Further high quality research reporting HSUVs for health states of controlled remission, and relapsed or refractory disease are also required. Additionally, future studies of HSUVs for older people with AML should be conducted in real-world settings rather than clinical trials to promote generalisability of the utility values.

Intensive therapy, low intensity therapy, and supportive care for people with AML are all resource intensive interventions [12, 13]. Major drivers of costs include hospitalisation and medication [14, 15]. Hospitalisation costs are likely to reduce over the coming decade as more treatments are offered in out-patient settings [14]. However, medication costs are expected to increase with the continued development of novel drugs [14]. Greater numbers of older people with AML who are unsuitable for intensive therapy will be eligible to undergo expensive novel targeted therapies that may prolong their life [14], increasing the costs of treatment of AML [13, 14]. It is vital that the value of such therapies relative to other options is rigorously evaluated to inform resource allocation decisions.

### Strengths and limitations

A strength of this review was the broad search strategy conducted across multiple databases, with detailed search terms, including studies of any date. The review was conducted with rigorous methods according to published guidelines. Additionally, the inclusion of HRQoL measures that can be converted to utility values with algorithms, reduced the likelihood of missing studies with relevant data. Forwards and backwards citation tracking, and cross referencing with previous reviews reduced any potential gaps in the search results. A limitation of this review was that only studies in English were included. It is unlikely many publications were missed as most (98%) scientific journals are published in English [65].

## Conclusions

Greater evidence is needed regarding HSUVs of older people with AML that can be used in economic evaluations, particularly for treatment-related health states in a real-world setting. Researchers should be encouraged and supported to publish HRQoL measures in sufficient detail to enable conversion to HSUVs, especially data derived from EORTC-QLQ-C30, which is a commonly used and appropriate tool for this population. Publishing all data would facilitate more rigorous economic evaluation in this important area.

## Electronic Supplementary Material

Below is the link to the electronic supplementary material.

Supplementary Material 1

Supplementary Material 2
