# Supplementary material for: A systematic review of health state utility values for older people with acute myeloid leukaemia
Source: Qual Life Res. 2024 Aug 22;33(11):2899–914. doi: 10.1007/s11136-024-03734-9 (PMC11541279; doi:10.1007/s11136-024-03734-9)
Supplement: Supplementary file 1 — Supplementary Material 1 [file 11136_2024_3734_MOESM1_ESM.docx]

**Supplementary materials**

Supplementary file 1. Combined database search terms

| **Search category** | **Search terms** |
| --- | --- |
| Acute myeloid leukaemia | AML OR Acute myelo* leuk* |
| Older (>60 years) | Older OR elderly |
| Utility terms | Utilit* or disutilit* OR HSUV OR “health state utility value”  “Quality adjusted life year*” or QALY or “quality-adjusted life year*” or “quality-adjusted life-year*” |
| Indirect valuation methods | EQ-5D or “EQ 5D” or EQ5D or Euroqol or “Euro qol” or EQ-5D-Y or "EQ 5D Y"  “European Organization for Research and Treatment of Cancer Quality-of-Life Questionnaire Core 30” OR “EORTC QLQ-C30”  “Short-form survey-6D” OR “short form 6D” OR SF-6D or “SF 6D” OR SF6D  “Short-form survey-12” OR “short form 12” OR “SF 12” or SF12  “Short-form survey-36” OR “short form 36” OR SF-36 or “SF 36” OR SF36  “Health utilities index” OR HUI OR HUI2 OR HUI3  “Quality of well being” OR “quality of well-being” OR “QWB  16D Health-Related Quality of Life” OR “16D HRQoL” OR “17D Health-Related Quality of Life” OR “17D HRQoL”  “Assessment of Quality of Life-6D” OR AQoL-6D OR “Assessment of Quality of Life-4D” AQoL-4D OR “Assessment of Quality of Life-8D” OR AQoL-8D  “Multi-attribute utility instrument” OR “multiattribute utility instrument” |
| Direct valuation methods | “Standard gamble” OR “standard-gamble”  “Time trade off” OR “time trade-off” OR TTO  “Best worst scaling” OR “best-worst scaling”  “Discrete choice experiment” OR “discrete-choice experiment” OR DCE  “Person trade off” OR “person trade-off”  “Scoring algorithm” OR “scoring-algorithm”  “Utility elicitation” OR “direct elicitation” |

Supplementary file 2. Individual database search terms

| **PubMed** | |
| --- | --- |
| # | Key words / limits |
| 1 | (AML[Title/Abstract] OR Acute myelo* leuk*[Title/Abstract]) OR ("leukemia, myeloid, acute"[MeSH Terms]) |
| 2 | (Older[Title/Abstract] OR elderly[Title/Abstract] OR geriatric[Title/Abstract]) OR ("Aged"[Mesh] OR "Frail Elderly"[Mesh]) |
| 3 | (Utilit*[Title/Abstract] OR disutilit*[Title/Abstract] OR HSUV[Title/Abstract] OR "health state utility value"[Title/Abstract] OR "Quality adjusted life year*"[Title/Abstract] OR QALY[Title/Abstract] OR "quality-adjusted life year*"[Title/Abstract] OR "quality-adjusted life-year*"[Title/Abstract]))  **OR**  ((EQ-5D[Title/Abstract] OR “EQ 5D”[Title/Abstract] OR EQ5D[Title/Abstract] OR EuroQol[Title/Abstract] OR "EORTC QLQ-C30"[Title/Abstract] OR "Short form"[Title/Abstract] OR "quality of well-being"[Title/Abstract] OR QWB[Title/Abstract] OR "health utilities index"[Title/Abstract] OR SF[Title/Abstract] OR AQoL[Title/Abstract] OR HRQoL[Title/Abstract] OR "multiattribute utility instrument"[Title/Abstract])  **OR**  ("Standard gamble"[Title/Abstract] OR "standard-gamble"[Title/Abstract] OR "Time trade off"[Title/Abstract] OR "time trade-off"[Title/Abstract] OR TTO[Title/Abstract] OR "Best worst scaling"[Title/Abstract] OR "best-worst scaling"[Title/Abstract] OR "Discrete choice experiment"[Title/Abstract] OR "discrete-choice experiment"[Title/Abstract] OR DCE[Title/Abstract] OR "Person trade off"[Title/Abstract] OR "person trade-off"[Title/Abstract] OR "Scoring algorithm"[Title/Abstract] OR "scoring-algorithm"[Title/Abstract] OR "Utility elicitation"[Title/Abstract] OR "direct elicitation"[Title/Abstract]) OR "Quality-Adjusted Life Years"[Majr] |
| 4 | 1 AND 2 AND 3 |
| **EMBASE** | |
| 1 | 'aml'/exp OR aml OR acute) AND myelo* AND leuk* |
| 2 | old OR 'elderly'/exp OR elderly OR 'geriatric'/exp OR geriatric |
| 3 | (utilit* OR disutilit* OR hsuv OR 'health state utility value') AND 'quality adjusted life year*' OR qaly OR 'quality-adjusted life year*' OR 'quality-adjusted life-year*'  OR  'eq 5d' OR eq5d OR euroqol OR 'eortc qlq-c30' OR 'eortc qlq c30' OR 'qlq c30' OR 'short form' OR 'quality of well-being' OR qwb OR 'health utilities index' OR sf OR aqol OR hrqol OR 'multiattribute utility instrument'  OR  'standard gamble' OR 'standard-gamble' OR 'time trade off' OR 'time trade-off' OR tto OR 'best worst scaling' OR 'best-worst scaling' OR 'discrete choice experiment' OR 'discrete-choice experiment' OR dce OR 'person trade off' OR 'person trade-off' OR 'scoring algorithm' OR 'scoring-algorithm' OR 'utility elicitation' OR 'direct elicitation' |
| 4 | 1 AND 2 AND 3 |
| **CINAHL** | |
| 1 | TI ( AML OR Acute myelo* leuk* ) OR AB ( AML OR Acute myelo* leuk* ) OR MW ( AML OR Acute myelo* leuk* ) OR (MH "Leukemia, Myeloid, Acute+") |
| 2 | TI ( older adults OR elderly OR seniors or geriatrics ) OR AB ( older adults OR elderly OR seniors or geriatrics ) OR MW ( older adults OR elderly OR seniors or geriatrics ) OR (MH "Older Adult Care (Saba CCC)") OR (MH "Frail Elderly") OR (MH "Aged+") |
| 3 | TI ( Utilit* OR disutilit* OR HSUV OR “health state utility value” OR “Quality adjusted life year*” OR QALY or “quality-adjusted life year*” OR “quality-adjusted life-year*” ) OR AB ( Utilit* OR disutilit* OR HSUV OR “health state utility value” OR “Quality adjusted life year*” OR QALY or “quality-adjusted life year*” OR “quality-adjusted life-year*” ) OR MW ( Utilit* OR disutilit* OR HSUV OR “health state utility value” OR “Quality adjusted life year*” OR QALY or “quality-adjusted life year*” OR “quality-adjusted life-year*” ) OR (MH "Quality-Adjusted Life Years")  **OR**  TI ( EQ-5D OR "EQ 5D" OR EQ5D OR EuroQol OR "EORTC QLQ-C30" OR "EORTC QLQ C30" OR QLQ-C30 OR "Short form" OR "quality of well-being" OR QWB OR "health utilities index" OR SF OR AQoL OR HRQoL OR "multiattribute utility instrument" ) OR AB ( EQ-5D OR "EQ 5D" OR EQ5D OR EuroQol OR "EORTC QLQ-C30" OR "EORTC QLQ C30" OR QLQ-C30 OR "Short form" OR "quality of well-being" OR QWB OR "health utilities index" OR SF OR AQoL OR HRQoL OR "multiattribute utility instrument" ) OR MW ( EQ-5D OR "EQ 5D" OR EQ5D OR EuroQol OR "EORTC QLQ-C30" OR "EORTC QLQ C30" OR QLQ-C30 OR "Short form" OR "quality of well-being" OR QWB OR "health utilities index" OR SF OR AQoL OR HRQoL OR "multiattribute utility instrument" )  **OR**  TI ( “Standard gamble” OR “standard-gamble” OR “Time trade off” OR “time trade-off” OR TTO OR “Best worst scaling” OR “best-worst scaling” OR “Discrete choice experiment” OR “discrete-choice experiment” OR DCE OR “Person trade off” OR “person trade-off” OR “Scoring algorithm” OR “scoring-algorithm” OR “Utility elicitation” OR “direct elicitation” ) OR AB ( “Standard gamble” OR “standard-gamble” OR “Time trade off” OR “time trade-off” OR TTO OR “Best worst scaling” OR “best-worst scaling” OR “Discrete choice experiment” OR “discrete-choice experiment” OR DCE OR “Person trade off” OR “person trade-off” OR “Scoring algorithm” OR “scoring-algorithm” OR “Utility elicitation” OR “direct elicitation” ) OR MW ( “Standard gamble” OR “standard-gamble” OR “Time trade off” OR “time trade-off” OR TTO OR “Best worst scaling” OR “best-worst scaling” OR “Discrete choice experiment” OR “discrete-choice experiment” OR DCE OR “Person trade off” OR “person trade-off” OR “Scoring algorithm” OR “scoring-algorithm” OR “Utility elicitation” OR “direct elicitation” ) |
| 4 | 1 AND 2 AND 3 |
| **PsycINFO** | |
| 1 | TI ( AML OR Acute myelo* leuk* ) OR AB ( AML OR Acute myelo* leuk* ) OR MW ( AML OR Acute myelo* leuk* ) |
| 2 | TI ( older adults OR elderly OR seniors or geriatrics ) OR AB ( older adults OR elderly OR seniors or geriatrics ) OR MW ( older adults OR elderly OR seniors or geriatrics ) OR DE "Geriatric Patients" |
| 3 | TI ( Utilit* OR disutilit* OR HSUV OR “health state utility value” OR “Quality adjusted life year*” OR QALY or “quality-adjusted life year*” OR “quality-adjusted life-year*” ) OR AB ( Utilit* OR disutilit* OR HSUV OR “health state utility value” OR “Quality adjusted life year*” OR QALY or “quality-adjusted life year*” OR “quality-adjusted life-year*” ) OR MW ( Utilit* OR disutilit* OR HSUV OR “health state utility value” OR “Quality adjusted life year*” OR QALY or “quality-adjusted life year*” OR “quality-adjusted life-year*” ) OR DE "Quality of Life" OR DE "Health Related Quality of Life" OR DE "Quality of Work Life" OR DE "Quality of Life Measures"  **OR**  TI ( EQ-5D OR "EQ 5D" OR EQ5D OR EuroQol OR "EORTC QLQ-C30" OR "EORTC QLQ C30" OR QLQ-C30 OR "Short form" OR "quality of well-being" OR QWB OR "health utilities index" OR SF OR AQoL OR HRQoL OR "multiattribute utility instrument" ) OR AB ( EQ-5D OR "EQ 5D" OR EQ5D OR EuroQual OR "EORTC QLQ-C30" OR "EORTC QLQ C30" OR QLQ-C30 OR "Short form" OR "quality of well-being" OR QWB OR "health utilities index" OR SF OR AQoL OR HRQoL OR "multiattribute utility instrument" ) OR MW ( EQ-5D OR "EQ 5D" OR EQ5D OR EuroQol OR "EORTC QLQ-C30" OR "EORTC QLQ C30" OR QLQ-C30 OR "Short form" OR "quality of well-being" OR QWB OR "health utilities index" OR SF OR AQoL OR HRQoL OR "multiattribute utility instrument" )  **OR**  TI ( “Standard gamble” OR “standard-gamble” OR “Time trade off” OR “time trade-off” OR TTO OR “Best worst scaling” OR “best-worst scaling” OR “Discrete choice experiment” OR “discrete-choice experiment” OR DCE OR “Person trade off” OR “person trade-off” OR “Scoring algorithm” OR “scoring-algorithm” OR “Utility elicitation” OR “direct elicitation” ) OR AB ( “Standard gamble” OR “standard-gamble” OR “Time trade off” OR “time trade-off” OR TTO OR “Best worst scaling” OR “best-worst scaling” OR “Discrete choice experiment” OR “discrete-choice experiment” OR DCE OR “Person trade off” OR “person trade-off” OR “Scoring algorithm” OR “scoring-algorithm” OR “Utility elicitation” OR “direct elicitation” ) OR MJ ( “Standard gamble” OR “standard-gamble” OR “Time trade off” OR “time trade-off” OR TTO OR “Best worst scaling” OR “best-worst scaling” OR “Discrete choice experiment” OR “discrete-choice experiment” OR DCE OR “Person trade off” OR “person trade-off” OR “Scoring algorithm” OR “scoring-algorithm” OR “Utility elicitation” OR “direct elicitation” ) |
| 4 | 1 AND 2 AND 3 |
| 5 | 1 AND 3 |
| **Cochrane Central** | |
| 1 | AML OR Acute myelo* leuk* (Title, abstract, keyword) |
| 2 | Older OR elderly OR geriatric (Title, abstract, keyword) |
| 3 | Utilit* OR disutilit* OR HSUV OR health state utility value OR Quality adjusted life year* OR QALY or quality-adjusted life year* OR quality-adjusted life-year* (Title, abstract, keyword)  **OR**  EQ-5D OR EQ 5D OR EQ5D OR EuroQol OR EORTC QLQ-C30 OR Short form OR quality of well-being OR QWB OR health utilities index OR SF OR AQoL OR HRQoL OR multiattribute utility instrument (Title, abstract, keyword)  **OR**  Standard gamble OR standard-gamble OR Time trade off OR time trade-off OR TTO OR Best worst scaling OR best-worst scaling OR Discrete choice experiment OR discrete-choice experiment OR DCE OR Person trade off OR person trade-off OR Scoring algorithm OR scoring-algorithm OR Utility elicitation OR direct elicitation (Title, abstract, keyword) |
| 4 | 1 AND 2 AND 3 |
| **EconLit** | |
| 1 | TI ( AML OR Acute myelo* leuk* ) OR AB ( AML OR Acute myelo* leuk* ) OR SU ( AML OR Acute myelo* leuk* ) |
| 2 | TI ( Older OR elderly OR geriatric ) OR AB ( Older OR elderly OR geriatric ) OR SU ( Older OR elderly OR geriatric ) |
| 3 | TI ( Utilit* OR disutilit* OR HSUV OR “health state utility value” OR “Quality adjusted life year*” OR QALY or “quality-adjusted life year*” OR “quality-adjusted life-year*” ) OR AB ( Utilit* OR disutilit* OR HSUV OR “health state utility value” OR “Quality adjusted life year*” OR QALY or “quality-adjusted life year*” OR “quality-adjusted life-year*” ) OR SU ( Utilit* OR disutilit* OR HSUV OR “health state utility value” OR “Quality adjusted life year*” OR QALY or “quality-adjusted life year*” OR “quality-adjusted life-year*” )  **OR**  TI ( EQ-5D OR EuroQol OR EORTC QLQ-C30 OR Short form OR quality of well-being OR QWB OR health utilities index OR SF OR AQoL OR HRQoL OR multiattribute utility instrument ) OR AB ( EQ-5D OR EuroQol OR EORTC QLQ-C30 OR Short form OR quality of well-being OR QWB OR health utilities index OR SF OR AQoL OR HRQoL OR multiattribute utility instrument ) OR SU ( EQ-5D OR EuroQol OR EORTC QLQ-C30 OR Short form OR quality of well-being OR QWB OR health utilities index OR SF OR AQoL OR HRQoL OR multiattribute utility instrument )  **OR**  TI ( "Standard gamble" OR "standard-gamble" OR "Time trade off" OR "time trade-off" OR TTO OR "Best worst scaling" OR "best-worst scaling" OR "Discrete choice experiment" OR "discrete-choice experiment" OR DCE OR "Person trade off" OR "person trade-off" OR "Scoring algorithm" OR "scoring-algorithm" OR "Utility elicitation" OR "direct elicitation" ) OR AB ( "Standard gamble" OR "standard-gamble" OR "Time trade off" OR "time trade-off" OR TTO OR "Best worst scaling" OR "best-worst scaling" OR "Discrete choice experiment" OR "discrete-choice experiment" OR DCE OR "Person trade off" OR "person trade-off" OR "Scoring algorithm" OR "scoring-algorithm" OR "Utility elicitation" OR "direct elicitation" ) OR SU ( "Standard gamble" OR "standard-gamble" OR "Time trade off" OR "time trade-off" OR TTO OR "Best worst scaling" OR "best-worst scaling" OR "Discrete choice experiment" OR "discrete-choice experiment" OR DCE OR "Person trade off" OR "person trade-off" OR "Scoring algorithm" OR "scoring-algorithm" OR "Utility elicitation" OR "direct elicitation" ) |
| 4 | 1 AND 2 AND 3 |
| 5 | 1 AND 3 |
|  | **Global Health** |
| 1 | TI ( AML OR Acute myelo* leuk* ) OR AB ( AML OR Acute myelo* leuk* ) OR SU ( AML OR Acute myelo* leuk* ) |
| 2 | TI ( Older OR elderly OR geriatric ) OR AB ( Older OR elderly OR geriatric ) OR SU ( Older OR elderly OR geriatric ) |
| 3 | TI ( Utilit* OR disutilit* OR HSUV OR “health state utility value” OR “Quality adjusted life year*” OR QALY or “quality-adjusted life year*” OR “quality-adjusted life-year*” ) OR AB ( Utilit* OR disutilit* OR HSUV OR “health state utility value” OR “Quality adjusted life year*” OR QALY or “quality-adjusted life year*” OR “quality-adjusted life-year*” ) OR SU ( Utilit* OR disutilit* OR HSUV OR “health state utility value” OR “Quality adjusted life year*” OR QALY or “quality-adjusted life year*” OR “quality-adjusted life-year*” )  **OR**  TI ( EQ-5D OR EuroQol OR EORTC QLQ-C30 OR Short form OR quality of well-being OR QWB OR health utilities index OR SF OR AQoL OR HRQoL OR multiattribute utility instrument ) OR AB ( EQ-5D OR EuroQol OR EORTC QLQ-C30 OR Short form OR quality of well-being OR QWB OR health utilities index OR SF OR AQoL OR HRQoL OR multiattribute utility instrument ) OR SU ( EQ-5D OR EuroQol OR EORTC QLQ-C30 OR Short form OR quality of well-being OR QWB OR health utilities index OR SF OR AQoL OR HRQoL OR multiattribute utility instrument )  **OR**  TI ( "Standard gamble" OR "standard-gamble" OR "Time trade off" OR "time trade-off" OR TTO OR "Best worst scaling" OR "best-worst scaling" OR "Discrete choice experiment" OR "discrete-choice experiment" OR DCE OR "Person trade off" OR "person trade-off" OR "Scoring algorithm" OR "scoring-algorithm" OR "Utility elicitation" OR "direct elicitation" ) OR AB ( "Standard gamble" OR "standard-gamble" OR "Time trade off" OR "time trade-off" OR TTO OR "Best worst scaling" OR "best-worst scaling" OR "Discrete choice experiment" OR "discrete-choice experiment" OR DCE OR "Person trade off" OR "person trade-off" OR "Scoring algorithm" OR "scoring-algorithm" OR "Utility elicitation" OR "direct elicitation" ) OR SU ( "Standard gamble" OR "standard-gamble" OR "Time trade off" OR "time trade-off" OR TTO OR "Best worst scaling" OR "best-worst scaling" OR "Discrete choice experiment" OR "discrete-choice experiment" OR DCE OR "Person trade off" OR "person trade-off" OR "Scoring algorithm" OR "scoring-algorithm" OR "Utility elicitation" OR "direct elicitation" ) |
| 4 | 1 AND 2 AND 3 |
| 5 | 1 AND 3 |
| **ProQuest Theses and Dissertations Database** | |
| 1 | [AML OR “Acute myelo* leuk*](https://www.proquest.com/recentsearches.recentsearchtabview.recentsearchesgridview.scrolledrecentsearchlist.checkdbssearchlink:rerunsearch/1E18158118794B1EPQ/None?site=pqdtglobal&t:ac=RecentSearches)” |
| 2 | "utility values" OR "health state utility value" OR "quality of life" OR EQ-5D OR "EORTC QLQ-C30" |
| 3 | geriatric OR "older people" |
| 4 | 1 AND 2 AND 3 |
| 5 | English only |
| 6 | In Abstract, Title or Subject Heading |

Supplementary file 3. Calculations for mapping EORTC-QLQ-C30 to EQ-5D

| **Domain from EORTC-QLQ-C30** | **Kim’s algorithm** | **Values** |
| --- | --- | --- |
| **Lennmyr 2020^25^** |  |  |
| Constant | 0.56317 | 0.56317 |
| *Global health status* | 0.00097*64.65 | 0.0627105 |
| *Physical functioning* | 0.00222*73.48 | 0.1631256 |
| *Role functioning* | 0.00067*49.29 | 0.0330243 |
| *Emotional functioning* | 0.00045*78.3 | 0.035235 |
| *Pain* | -0.00125*18.25 | -0.0228125 |
|  | Utility value (sum) | **0.8344529** |
| **Pierson 2017^26^** |  |  |
| **All patients** |  |  |
| Constant | 0.56317 | 0.56317 |
| *Global health status* | 0.00097*50.1 | 0.048597 |
| *Physical functioning* | 0.00222*67.6 | 0.150072 |
| *Role functioning* | 0.00067*62.1 | 0.041607 |
| *Emotional functioning* | 0.00045*71.6 | 0.03222 |
| *Pain* | -0.00125*18.8 | -0.0235 |
|  | Utility value (sum) | **0.812166** |
| **ECOG Performance Status 0** |  |  |
| Constant | 0.56317 | 0.56317 |
| *Global health status* | 0.00097*65.2 | 0.063244 |
| *Physical functioning* | 0.00222*80.9 | 0.179598 |
| *Role functioning* | 0.00067*79.1 | 0.052997 |
| *Emotional functioning* | 0.00045*77.8 | 0.03501 |
| *Pain* | -0.00125*9.3 | -0.011625 |
|  | Utility value (sum) | **0.882394** |
| **ECOG Performance Status 1** |  |  |
| Constant | 0.56317 | 0.56317 |
| *Global health status* | 0.00097*48.5 | 0.047045 |
| *Physical functioning* | 0.00222*69.4 | 0.154068 |
| *Role functioning* | 0.00067*63.2 | 0.042344 |
| *Emotional functioning* | 0.00045*72.8 | 0.03276 |
| *Pain* | -0.00125*17.1 | -0.021375 |
|  | Utility value (sum) | **0.818012** |
| **ECOG Performance Status 1** |  |  |
| Constant | 0.56317 | 0.56317 |
| *Global health status* | 0.00097*43.1 | 0.041807 |
| *Physical functioning* | 0.00222*54.4 | 0.120768 |
| *Role functioning* | 0.00067*48.2 | 0.032294 |
| *Emotional functioning* | 0.00045*65.0 | 0.02925 |
| *Pain* | -0.00125*28.9 | -0.036125 |
|  | Utility value (sum) | **0.751164** |

ECOG – Eastern Oncology Outcomes Group

Supplementary file 4. Studies excluded after full-text review

| **Excluded studies** | | **Reason excluded** |
| --- | --- | --- |
| 1 | Alati, C.; Ronco, F.; Candoni, A.; Di Bartolomeo, P.; Simeone, E.; Freyrie, A.; Volpe, A.; Musto, P.; Cascavilla, N.; Capelli, D.; Di Raimondo, F.; Niscola, P.; Cortelezzi, A.; Salutari, P.; Oliva, E. N. Quality of life at diagnosis in elderly patients with acute myeloid leukemia considered fit for induction of remission chemotherapy. Haematologica 2015;100:36. | HSUV not described in enough detail |
| 2 | Alibhai, S. M.; O'Neill, S.; Fisher-Schlombs, K.; Breunis, H.; Timilshina, N.; Brandwein, J. M.; Minden, M. D.; Tomlinson, G. A.; Culos-Reed, S. N. A pilot phase II RCT of a home-based exercise intervention for survivors of AML Supportive Care in Cancer 2014;22(4):881‐889. | HSUV not described in enough detail |
| 3 | Batty, N.; Wiles, S.; Kabalan, M.; Sharma, R.; Shatzel, J.; Pang, J.; Yi, D.; Alatovic, I.; Saif, S.; Narasimha, D.; La Penna, J.; Troitino, A.; Attwood, K.; Yin, Y.; Wetzler, M. Decitabine is more cost effective than standard conventional induction therapy in elderly acute myeloid leukemia patients. Blood 2013;122(21). | HSUV not described in enough detail |
| 4 | Bewersdorf, J. P.; Patel, K. K.; Goshua, G.; Shallis, R. M.; Podoltsev, N. A.; Stahl, M.; Stein, E. M.; Huntington, S. F.; Zeidan, A. M. 2022. Cost-effectiveness of azacitidine and ivosidenib in newly diagnosed older, intensive chemotherapy-ineligible patients with IDH1-mutant acute myeloid leukemia. Leuk Lymphoma Dec 9 2022;1-8. | HSUV not described in enough detail |
| 5 | Bell, J. A.; Pompilus, F. A.; Rams, A.; Zhu, Y.; Ciesluk, A.; Bejar, R.; Fram, R. J.; Faller, D. V.; Marquis, P. Patient-centered evaluation of clinical benefit in acute myeloid leukemia: Importance of early engagement with patients. Blood 2019;134. | Not primary HSUV data reported |
| 6 | Bewersdorf, J. P.; Goshua, G.; Patel, K. K.; Shallis, R. M.; Podoltsev, N.; Huntington, S. F.; Zeidan, A. M. Cost-Effectiveness of Liposomal Cytarabine-Daunorubicin (CPX-351) Compared to Conventional Cytarabine-Daunorubicin Chemotherapy in Acute Myeloid Leukemia Blood 2021;138:11. | Not primary HSUV data reported |
| 7 | Castejón, N.; Cappelleri, J. C.; Cuervo, J.; Lang, K.; Mehta, P.; Mokgokong, R.; Mamolo, C. Social preferences for health states associated with acute myeloid leukemia for patients undergoing treatment in the United Kingdom Health Qual Life Outcomes Apr 18 2018;16(1):66. | Wrong patient population |
| 8 | Cheng, M. J.; Smith, B. D.; Hourigan, C. S.; Gojo, I.; Pratz, K. W.; Blackford, A. L.; Mehta, A. K.; Smith, T. J. A Single Center Survey of Health-Related Quality of Life among Acute Myeloid Leukemia Survivors in First Complete Remission. J Palliat Med Nov 2017;20(11):1267-1273. | Wrong patient population |
| 9 | Copland, M.; Ariti, C.; Thomas, I.; Upton, L.; Sydenham, M.; Mehta, P.; Islam, S.; Kjeldsen, L.; Burnett, A. K.; Hills, R.; Russell, N. H.; Dennis, M. A Randomised Evaluation of Low-Dose Cytarabine Arabinoside Plus Lenalidomide Versus Single-Agent Low-Dose Cytarabine Arabinoside in Older Patients with Acute Myeloid Leukaemia: Results from the LI-1 Trial. Blood 2021;138:1266. | HSUV not described in enough detail |
| 10 | Coyle, D.; Villeneuve, P. J. A. Economic Evaluation of Azacitidine in Elderly Patients with Acute Myeloid Leukemia with High Blast Counts. Pharmacoecon Open Jun 2020;4(2):297-305. | Not primary HSUV data reported |
| 11 | Deschler, B.; Ihorst, G.; Platzbecker, U.; Germing, U.; Lübbert, M. Development of a frailty score for older patients with myelodysplastic syndromes and acute myeloid Leukemia. Blood 2009;114(22). | HSUV not described in enough detail |
| 12 | Deschler, B.; Ihorst, G.; Schnitzler, S.; Bertz, H.; Finke, J. Geriatric assessment and quality of life in older patients considered for allogeneic hematopoietic cell transplantation: a prospective risk factor and serial assessment analysis. Bone Marrow Transplant May 2018;53(5):565-575. | HSUV not described in enough detail |
| 13 | Dohner, H.; Montesinos, P.; Polo, S. V.; Zarzycka, E.; Wang, J.; Bertani, G.; Heuser, M.; Calado, R. T.; Schuh, A. C.; Yeh, S. P.; de la Fuente, A.; Cerchione, C.; Daigle, S. R.; Hui, J.; Pandya, S. S.; Gianolio, D. A.; Recher, C.; de Botton, S. 2022. AML-295 AGILE: A Global, Randomized, Double-Blind, Phase 3 Study of Ivosidenib + Azacitidine Versus Placebo + Azacitidine in Patients With Newly Diagnosed Acute Myeloid Leukemia With an IDH1 Mutation. Clinical Lymphoma, Myeloma and Leukemia 2022;22:S234. | Wrong outcomes |
| 14 | Efficace, Fabio; Cottone, Francesco; Sommer, Kathrin; Kieffer, Jacobien; Aaronson, Neil; Fayers, Peter; Groenvold, Mogens; Caocci, Giovanni; Lo Coco, Francesco; Gaidano, Gianluca; Niscola, Pasquale; Baccarani, Michele; Rosti, Gianantonio; Venditti, Adriano; Angelucci, Emanuele; Fazi, Paola; Vignetti, Marco; Giesinger, Johannes. Validation of the European Organisation for Research and Treatment of Cancer Quality of Life Questionnaire Core 30 Summary Score in Patients With Hematologic Malignancies. Value in Health 2019;22(11):1303-1310. | Wrong patient population |
| 15 | Gan, G. G.; Ng, D. L. C.; Leong, Y. C.; Bee, P. C.; Chin, E. F. M.; Halim, H. A.; Aziz, T. A. H. T.; Loo, H.; Teh, A. K. H. Anxiety and depression in patients with haematological neoplasms in Malaysia. Medical Journal of Malaysia 2019;74(3):191-197. | Wrong patient population |
| 16 | Goswami, P.; Oliva, E. N.; Ionova, T.; Else, R.; Kell, J.; Fielding, A. K.; Jennings, D. M.; Karakantza, M.; Al-Ismail, S.; Collins, G. P.; McConnell, S.; Langton, C.; Al-Obaidi, M. J.; Oblak, M.; Salek, S. Hematological Malignancy Specific Patient-Reported Outcome Measure (HM-PRO): Construct Validity Study. Frontiers in Pharmacology 2020;11. | Wrong patient population |
| 17 | Guinan, K.; Mathurin, K.; Au, Y.; Schuh, A. C.; Bui, C. N.; Chai, X.; Lachaine, J. 2022. Venetoclax in Combination with Azacitidine for the Treatment of Newly Diagnosed Acute Myeloid Leukemia: A Canadian Cost-Utility Analysis. Curr Oncology, 29(10):7524-7536. | HSUV not described in enough detail |
| 18 | He, J.; Pierson, R.; Loefgren, C.; Cella, D. Patient-reported outcomes validation of the fact-LEU in acute myeloid leukemia: A review of baseline characteristics in AML2002. Blood 2018;132. | HSUV not described in enough detail |
| 19 | Hsu, Chiun; Wang, Jung-Der; Hwang, Jing-Shiang; Tien, Hwei-Fang; Chang, Shueh-Mei; Cheng, Ann-Lii; Chen, Yao-Chang; Tang, Jih-Luh. Survival-weighted health profile for long-term survivors of acute myelogenous leukemia. Quality of Life Research: An International Journal of Quality of Life Aspects of Treatment, Care & Rehabilitation 2003;12(5):503-517. | Wrong patient population |
| 20 | Hupfer, V.; Schmoor, C.; Schlenk, R. F.; Salih, H. R.; Crysandt, M.; Heuser, M.; Lindemann, H. W.; Muller-Tidow, C.; Bug, G.; Germing, U.; et al., Role of functional parameters in elderly AML patients deemed unfit for induction chemotherapy: results from the decider trial. Hemasphere 2020;4:796‐797. | HSUV not described in enough detail |
| 21 | Joshi, N.; Hensen, M.; Patel, S.; Xu, W.; Lasch, K.; Stolk, E. Health State Utilities for Acute Myeloid Leukaemia: A Time Trade-off Study. PharmacoEconomics 2019;37(1):85-92. | Wrong patient population |
| 22 | Kansal, A.; Du, M.; Herrera-Restrepo, O.; Leipold, R.; Ryan, R. J.; Louie, A. C.; Chung, K. Cost-effectiveness of CPX-351 versus 7+3 regimen in the treatment of treatment-related acute myeloid leukemia (TAML) or aml with myelodysplasia-related changes (MRC). Blood 2017;130. | HSUV not described in enough detail |
| 23 | Kornblith AB, Herndon JE II, Silverman LR, et al. Impact of azacytidine on the quality of life of patients with myelodysplastic syndrome treated in a randomized phase III trial: a Cancer and Leukemia Group B study. J Clin Oncol. 2002;20(10):2441-2452. | Wrong population |
| 24 | Kurosawa, S.; Yamaguchi, T.; Mori, T.; Kanamori, H.; Onishi, Y.; Emi, N.; Fujisawa, S.; Kohno, A.; Nakaseko, C.; Saito, B.; Kondo, T.; Hino, M.; Nawa, Y.; Kato, S.; Hashimoto, A.; Fukuda, T. Patient-reported quality of life after allogeneic hematopoietic cell transplantation or chemotherapy for acute leukemia. Bone Marrow Transplant Sep 2015;50(9):1241-9. | Wrong patient population |
| 25 | Leunis, A.; Redekop, W. K.; Uyl-de Groot, C. A.; Löwenberg, B. Impaired health-related quality of life in acute myeloid leukemia survivors: a single-center study. Eur J Haematol Sep 2014;93(3):198-206. | Wrong patient population |
| 26 | Levy, AR; Zou, D; Risebrough, N; Buckstein, R; Kim, T; Brereton, N. Cost-effectiveness in Canada of azacitidine for the treatment of higher-risk myelodysplastic syndromes. Current Oncology 2014;21(1):e29. | Not primary HSUV data reported |
| 27 | Lowe, Jared R.; Yu, Yinxi; Wolf, Steven; Samsa, Greg; LeBlanc, Thomas W. A cohort study of patient-reported outcomes and healthcare utilization in acute myeloid leukemia patients receiving active cancer therapy in the last six months of life. Journal of Palliative Medicine 2018;21(5):592-597. | Wrong outcomes |
| 28 | Lymanets, T.; Skrypnyk, I.; Maslova, G.; Gusachenko, I. Quality of life in acute leukemia patients with comorbid ischemic heart disease. Hemasphere 2018;2:452. | Wrong patient population |
| 29 | Mareque, M.; Montesinos, P.; Font, P.; Guinea, J. M.; de la Fuente, A.; Soto, J.; Oyagüez, I.; Brockbank, J.; Iglesias, T.; Llinares, J.; Sierra, J. Cost-effectiveness analysis of gemtuzumab ozogamicin for first-line treatment of patients with cd-33 positive acute myeloid leukaemia in Spain. ClinicoEconomics and Outcomes Research 2021;13():263-277. | Not primary HSUV data reported |
| 30 | Matza, L. S.; Deger, K. A.; Howell, T. A.; Koetter, K.; Yeager, A. M.; Hogge, D.; Fisher, V.; Louie, A. C.; Chung, K. C. Health state utilities associated with treatment options for acute myeloid leukemia (AML). J Med Econ Jun 2019;22(6):567-576. | Wrong patient population |
| 31 | Moller, T.; Adamsen, L.; Appel, C.; Welinder, P.; Stage, M.; Jarden, M.; Hjerming, M.; Kjeldsen, L. Health related quality of life and impact of infectious comorbidity in outpatient management of patients with acute leukemia. Leukemia and Lymphoma 2012;53(10):1896-1904. | Wrong patient population |
| 32 | Morishita, S.; Kaida, K.; Ikegame, K.; Yoshihara, S.; Taniguchi, K.; Okada, M.; Kodama, N.; Ogawa, H.; Domen, K. Impaired physiological function and health-related QOL in patients before hematopoietic stem-cell transplantation. Supportive care in cancer 2012;20(4):821-829. | Wrong patient population |
| 33 | Nordmann, P.; Schaffner, A.; Dazzi, H. Cost effectiveness in treatment of acute myeloid leukemia. Schweiz Med Wochenschr Dec 23 2000;130(51-52):1994-2000. | HSUV not described in enough detail |
| 34 | Oliva, E. N.; Candoni, A.; Salutari, P.; Di Raimondo, F.; Reda, G.; Capelli, D.; Niscola, P.; Selleri, C.; Musto, P.; Vigna, E.; et al., Azacitidine for post-remission therapy in elderly patients with acute myeloid leukemia: final results of the qoless AZA-Amle randomized trial. Blood 2019;134. | HSUV not described in enough detail |
| 35 | Oliva, E. N.; Nobile, F.; Alimena, G.; Ronco, F.; Specchia, G.; Impera, S.; Breccia, M.; Vincelli, I.; Carmosino, I.; Guglielmo, P.; Pastore, D.; Alati, C.; Latagliata, R. Quality of life in elderly patients with acute myeloid leukemia: Patients may be more accurate than physicians. Haematologica 2011;96(5):696-702. | HSUV not described in enough detail |
| 36 | Oliva, E. N.; Salutari, P.; Di Raimondo, F.; Reda, G.; Capelli, D.; Iannì, G.; Tripepi, G.; Alati, C.; Mammì, C.; D'Errigo, M. G.; Niscola, P.; Selleri, C.; Musto, P.; Vigna, E.; Volpe, A.; Cascavilla, N.; Cannatà, M. C.; Mannina, D.; Candoni, A. 2022. Final results of the QOLESS AZA-AMLE randomized trial to evaluate the efficacy of 5-AZA for post-remission therapy of acute myeloid leukaemia in elderly patients. HemaSphere 2022;6:892-893. | HSUV not described in enough detail |
| 37 | Oliva, E. N.; Salutari, P.; Candoni, A.; Freyrie, A.; Capelli, D.; Di Raimondo, F.; Volpe, A.; Cascavilla, N.; Di Bartolomeo, P.; Simeone, E.; Cortelezzi, A.; Leoni, P.; Musto, P.; Morabito, F.; Niscola, P.; Ranieri, N.; Santacaterina, I.; Marino, A. G.; Cufari, P.; Alati, C.; Ronco, F. Quality of life in elderly patients with acute myeloid leukemia undergoing induction chemotherapy.  Blood 2015;126(23):2120. | HSUV not described in enough detail |
| 38 | Patel, K. K.; Zeidan, A. M.; Shallis, R. M.; Prebet, T.; Podoltsev, N.; Huntington, S. F. Cost-effectiveness of azacitidine and venetoclax in unfit patients with previously untreated acute myeloid leukemia. Blood Adv Feb 23 2021;5(4):994-1002. | Not primary HSUV data reported |
| 39 | Pierson, R.; He, J.; Xiu, L.; Nemat, S.; Loefgren, C.; Thomas, X. Patient-reported disease burden in the elderly patients with acute myeloid leukemia. Blood 2017;130. | HSUV not described in enough detail |
| 40 | Pleyer, Lisa; Heibl, Sonja; Tinchon, Christoph; Vallet, Sonja; Petricevic, Branka; Leisch, Michael; Egle, Alexander; Melchardt, Thomas; Piringer, Gudrun; Wolf, Dominik. The EQ-5D-5L Predicts Treatment Outcomes and Provides Added Value to the R-IPSS in Patients with MDS, CMML or AML Treated within the Austrian Azacitidine Registry-a Prospective Cohort Study By the AGMT Study Group. Blood 2021;138:64. | Wrong patient population |
| 41 | Pratz, K. W.; Panayiotidis, P.; Recher, C.; Wei, X.; Jonas, B. A.; Montesinos, P.; Ivanov, V.; Schuh, A. C.; Dinardo, C. D.; Novak, J.; Pejsa, V.; Stevens, D. A.; Yeh, S. P.; Kim, I.; Turgut, M.; Fracchiolla, N.; Yamamoto, K.; Ofran, Y.; Wei, A. H.; Bui, C.; Benjamin, K.; Kamalakar, R.; Potluri, J.; Mendes, W.; Devine, J.; Fiedler, W. Delays in Time to Deterioration of Health-Related Quality of Life Were Observed in Patients with Acute Myeloid Leukemia Receiving Venetoclax in Combination with Azacitidine or in Combination with Low-Dose Cytarabine. Blood 2020;136:33-35. | HSUV not described in enough detail |
| 42 | Pratz, K. W.; Panayiotidis, P.; Recher, C.; Wei, X.; Jonas, B. A.; Montesinos, P.; Ivanov, V.; Schuh, A. C.; DiNardo, C. D.; Novak, J.; Pejsa, V.; Stevens, D.; Yeh, S. P.; Kim, I.; Turgut, M.; Fracchiolla, N.; Yamamoto, K.; Ofran, Y.; Wei, A. H.; Bui, C. N.; Benjamin, K.; Kamalakar, R.; Potluri, J.; Mendes, W.; Devine, J.; Fiedler, W. 2022. Venetoclax combinations delay the time to deterioration of HRQoL in unfit patients with acute myeloid leukemia. Blood Cancer Journal 2022;12(4). | HSUV not described in enough detail |
| 43 | Priscilla, Das; Hamidin, Awang; Azhar, Md Zain; Noorjan, Kon; Salmiah, Md Said; Bahariah, Khalid. The Socio-Demographic and Clinical Factors Associated with Quality of Life among Patients with Haematological Cancer in a Large Government Hospital in Malaysia. Malaysian Journal of Medical Sciences 2011;18(3):49-56. | Wrong patient population |
| 44 | Ramos, F.; Serrano, J.; Hermosin, L.; Fuertes-Núñez, M.; Martínez, P.; Rodríguez, C.; Barrios, M.; Ibañez, F.; Bernal, T.; Olave, M. T.; Álvarez Juárez, M. Á; Vahí, M.; Caballero-Velázquez, T.; González, B.; Altés, A.; Fernández, P.; Durán, M. A.; Barrenetxea Lekue, C.; López, R.; Rafel, M. Overall survival (OS) and quality of life (QOL) of older adults diagnosed with acute myeloid leukemia (AML) and treated under conditions of usual clinical practice: Interim report of the SVLMA study. Hemasphere 2020;4():233-234. | HSUV not described in enough detail |
| 45 | Richardson, D. R.; Crossnohere, N. L.; Seo, J.; Estey, E.; O'Donoghue, B.; Smith, B. D.; Bridges, J. F. P. Age at Diagnosis and Patient Preferences for Treatment Outcomes in AML: A Discrete Choice Experiment to Explore Meaningful Benefits. Cancer Epidemiol Biomarkers Prev May 2020;29(5):942-948. | Wrong patient population |
| 46 | Richardson, Daniel R.; Oakes, Allison H.; Crossnohere, Norah L.; Rathsmill, Gary; Reinhart, Crystal; O'Donoghue, Bernadette; Bridges, John F. P. Prioritizing the worries of AML patients: Quantifying patient experience using best–worst scaling. Psycho-Oncology 2021. | Wrong outcomes |
| 47 | Richardson, Daniel R.; Crossnohere, Norah L.; Cole, Amy C.; Adapa, Karthik; Teal, Randall; Khasawneh, Amro; Kwong, Elizabeth C.; Loh, Kah Poh; Mhina, Carl J.; Sorah, Jonathan D.; Bryant, Ashley Leak; Wheeler, Stephanie B.; Bridges, John F. P.; Wood, William Allen. 2022. Evaluating a patient-centered discrete choice experiment to quantify individual treatment preferences of newly diagnosed older adults with acute myeloid leukemia: A mixed methods study. Journal of Clinical Oncology 2022;40:256-256. | Wrong outcomes |
| 48 | Ritchie, E. K.; Cella, D.; Fabbiano, F.; Pigneux, A.; Kanda, Y.; Ivanescu, C.; Pandya, B. J.; Shah, M. V. The relationship between hospitalization and patient-reported outcomes (PROs) in patients with FLT3-mutated (FLT3mut+) relapsed/refractory (R/R) acute myeloid leukemia (AML): results from the phase 3 admiral study. Blood 2019;134. | HSUV not described in enough detail |
| 39 | Ritchie, E. K.; Klepin, H. D.; Storrick, E.; Major, B.; Le-Rademacher, J.; Wadleigh, M.; Walker, A.; Larson, R. A.; Roboz, G. J. 2022. Geriatric assessment for older adults receiving less-intensive therapy for acute myeloid leukemia: report of CALGB 361101. Blood Adv Jun 28 2022;6(12):3812-3820. | HSUV not described in enough detail |
| 50 | Roboz, G.; Dohner, H.; Pocock, C.; Dombret, H.; Ravandi, F.; Jang, J. H.; Selleslag, D.; Mayer, J.; Martens, U.; Liesveld, J.; Bernal, T.; Wang, M. C.; La Torre, I.; Skikne, B.; Kumar, K.; Dong, Q.; Braverman, J.; Abi Nehme, S.; Beach, C.; Wei, A. Health-related quality of life with CC-486 in patients with acute myeloid leukemia (AML) in first remission following induction chemotherapy (IC): Results from the phase iii Quazar AML-001 trial. Hemasphere 2020;4:128. | HSUV not described in enough detail |
| 51 | Roboz, G.J., et al., Health-related quality of life (HRQoL) in the phase III QUAZAR-AML-001 trial of CC-486 as maintenance therapy for patients with acute myeloid leukemia (AML) in first remission following induction chemotherapy (IC). 2020, American Society of Clinical Oncology. | HSUV not described in enough detail |
| 52 | Saillard, C.; Rousseau, F.; Cecile, M.; Braticevic, C.; Etienne, A.; Rey, J.; D'Incan, E.; Zemmour, C.; Vey, N.; Charbonnier, A. Evaluation of a standardized geriatric assessment at diagnosis in a prospective cohort of elderly patients with newly diagnosed acute myeloid leukemia. Blood 2018;132. | HSUV not described in enough detail |
| 53 | Sekeres MA, Stone RM, Zahrieh D, et al. Decision-making and quality of life in older adults with acute myeloid leukemia or advanced myelodysplastic syndrome. Leukemia 2004; 18:809–16. | Wrong population |
| 54 | Seo, J.; Smith, B. D.; Estey, E.; Voyard, E.; O' Donoghue B; Bridges, J. F. P. Developing an instrument to assess patient preferences for benefits and risks of treating acute myeloid leukemia to promote patient-focused drug development. Curr Med Res Opin Dec 2018;34(12):2031-2039. | Wrong patient population |
| 55 | Sierra, J.; Mareque, M.; Montesinos, P.; Guinea, J. M.; Font, P.; Oyagüez, I.; Brockbank, J.; Candini, D.; Llinares, J.; Soto, J.; De La Fuente, A. Cost-effectiveness of gemtuzumab ozogamicin in combination with standard of care chemotherapy for first-line treatment of patients with CD33-positive acute myeloid leukemia in Spain. Hemasphere 2020;4:795-796. | HSUV not described in enough detail |
| 56 | Sorror, M. L.; Storer, B. E.; Fathi, A. T.; Brunner, A. M.; Gerds, A. T.; Sekeres, M. A.; Mukherjee, S.; Medeiros, B. C.; Wang, E. S.; Vachhani, P.; et al., Multi-Site 11-Year Experience of Less-Intensive versus Intensive Therapies in Acute Myeloid Leukemia. Blood 2021. | HSUV not described in enough detail |
| 57 | Sorror, M. L.; Storer, B. E.; Gerds, A. T.; Medeiros, B. C.; Shami, P. J.; Galvin, J. P.; Adekola, K. U.; Luger, S.; Baer, M. R.; Rizzieri, D. A.; et al., Limitations to receiving allogeneic hematopoietic cell transplantation for treatment of acute myeloid leukemia: a large multi-center prospective longitudinal observational study. Blood 2018;132. | HSUV not described in enough detail |
| 58 | Stein, E. M.; Yang, M.; Guerin, A.; Gao, W.; Galebach, P.; Xiang, C. Q.; Bhattacharyya, S.; Bonifacio, G.; Joseph, G. J. Assessing utility values for treatment-related health states of acute myeloid leukemia in the United States. Health Qual Life Outcomes Sep 21 2018;16(1):193. | Wrong patient population |
| 59 | Tervonen, T.; Cutts, K.; Seo, J.; Nehme, S. A.; Torre, I. L.; Prawitz, T.; Chen, C.; Beach, C. L.; Wang, J. Patient preferences for maintenance treatment of acute myeloidleukemia: results of a discrete choice experiment. Blood 2020;136(SUPPL 1):38‐39. | HSUV not described in enough detail |
| 60 | Tremblay, G.; Dolph, M.; Sachin, Patel; Brandt, P.; Forsythe, A. Cost-effectiveness analysis for midostaurin versus standard of care in acute myeloid leukemia in the United Kingdom Cost Effectiveness and Resource Allocation // 2018;16(33):(4 October 2018). | Not primary HSUV data reported |
| 61 | Tremblay, Gabriel; Cariou, Clemence; Recher, Christian; Dolph, Mike; Brandt, Patricia; Blanc, Anne-Sandrine. Cost-Effectiveness of Midostaurin in the Treatment of Newly Diagnosed FLT3-Mutated Acute Myeloid Leukemia in France. European Journal of Health Economics 2020;21(4):543-555. | Not primary HSUV data reported |
| 62 | Van de Velde, A. L.; Beutels, P.; Smits, E. L.; Van Tendeloo, V. F.; Nijs, G.; Anguille, S.; Verlinden, A.; Gadisseur, A. P.; Schroyens, W. A.; Dom, S.; Cornille, I.; Goossens, H.; Berneman, Z. N. Medical costs of treatment and survival of patients with acute myeloid leukemia in Belgium. Leuk Res Jul 2016;46:26-9. | Wrong outcomes |
| 63 | Wang, C.; Yan, J.; Chen, J.; Wang, Y.; Lin, Y. C.; Hu, R.; Wu, Y. Factors associated with quality of life of adult patients with acute leukemia and their family caregivers in China: a cross-sectional study. Health Qual Life Outcomes Jan 7 2020;18(1):8. | Wrong patient population |
| 64 | Wang, E. S.; Heuser, M.; Montesinos, P.; Rich, E. S.; Wu, R.; Pandya, B. J.; Shah, M. V. Patient Reported Outcomes in Patients with Newly Diagnosed FLT3mut+ acute Myeloid Leukemia Ineligible for Intensive Induction Chemotherapy from Lacewing: A Randomized Phase 3 Trial of Gilteritinib and Azacitidine Versus Azacitidine Alone. Blood 2021;138:3058. | HSUV not described in enough detail |
| 65 | Wei, A. H.; Dohner, H.; Pocock, C.; Montesinos, P.; Afanasyev, B.; Dombret, H.; Ravandi, F.; Sayar, H.; Jang, J. H.; Porkka, K.; et al., The QUAZAR AML-001 maintenance trial: results of a phase III international, randomized, double-blind, placebo-controlled study of CC-486 (oral formulation of azacitidine) in patients with acute myeloid leukemia (AML) in first remission Blood 2019;134. | Wrong outcomes |
| 66 | Wettergren, L.; Sprangers, M.; Björkholm, M.; Langius-Eklöf, A. Quality of life before and one year following stem cell transplantation using an individualized and a standardized instrument. Psychooncology Apr 2008;17(4):338-46. | Wrong patient population |
| 67 | Wolach, Ofir; Levi, Itai; Canaani, Jonathan; Tadmor, Tamar; Tavor, Sigal; Hellmann, Ilana; Zuckerman, Tsila; Lavie, David; Stemer, Galia; Cohen, Raanan. First results from a nationwide prospective non-interventional study of venetoclax-based 1st line therapies in patients with acute myeloid leukemia (AML)-revive study. Blood 2020;136():27-28. | HSUV not described in enough detail |
| 67 | Yu, Hongjuan; Zeng, Xueyun; Sui, Mingjie; Liu, Rui; Tan, Rachel Lee-Yin; Yang, Jinjin; Huang, Weidong; Luo, Nan. A head-to-head comparison of measurement properties of the eq-5d-3l and eq-5d-5l in acute myeloid leukemia patients. Quality of Life Research: An International Journal of Quality of Life Aspects of Treatment, Care & Rehabilitation 2020. | Wrong patient population |
