# Supplementary material for: A systematic review of health state utility values for older people with acute myeloid leukaemia
Source: Qual Life Res. 2024 Aug 22;33(11):2899–914. doi: 10.1007/s11136-024-03734-9 (PMC11541279; doi:10.1007/s11136-024-03734-9)
Supplement: Supplementary file 2 — Supplementary Material 2 [file 11136_2024_3734_MOESM2_ESM.docx]

Table 7. Quality assessment of included studies using CASP (Critical Appraisal Skills Programme) checklist [14]

| **Full text studies** | | | | | | | | | | | | | |
| --- | --- | --- | --- | --- | --- | --- | --- | --- | --- | --- | --- | --- | --- |
| CASP – Economic evaluation checklist | Well defined research question | Description of competing alternatives | Evidence of effectiveness of intervention | Effects of the intervention identified, measured and valued | Relevant resources identified, measured and valued | Discounting performed | Results of evaluation adequately reported | Incremental analysis of the consequences and cost of alternatives | Adequate sensitivity analysis performed | Likely to be equally effective in your context or setting | Costs translatable to your setting | Worth doing in your setting |  |
| Groot 1998 [1] | 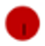 | 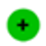 | 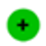 | 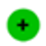 | 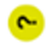 | 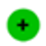 | 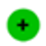 | 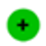 | 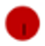 | 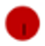 | 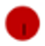 | 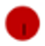 |  |
| CASP – Cohort study checklist | Clearly focused research question | Cohort recruited in an acceptable way | Exposure accurately measured to minimise bias | Outcome accurately measured to minimise bias | Important confounding factors identified | Confounding factors considered in design and/or analysis | Follow up of subjects complete, and long enough | Results of study adequately reported | Results reported with precision | Plausible results | Results applicable to local population | Results consistent with other evidence | Clear implications for practice |
| Mamolo 2019 [2] | 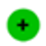 | 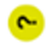 | 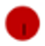 | 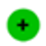 | 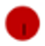 | 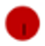 | 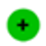 | 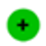 | 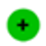 | 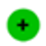 | 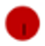 | 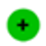 | 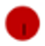 |
| Lennmyr  2020 [3] | 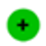 | 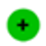 | 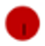 | 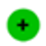 | 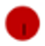 | 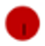 | 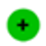 | 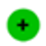 | 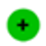 | 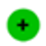 | 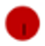 | 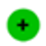 | 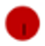 |
| Piepert, 2020 [5] | 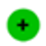 | 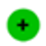 | 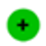 | 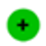 | 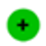 | 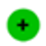 | 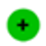 | 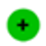 | 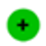 | 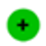 | 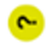 | 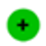 | 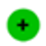 |

* CASP – Critical Appraisal Skills Programme / N.B. Insufficient information was reported in the included abstracts to conduct quality appraisal using the CASP tool
